# Effect of *Shuangdan Mingmu* capsule, a Chinese herbal formula, on oxidative stress-induced apoptosis of pericytes through PARP/GAPDH pathway

**DOI:** 10.1186/s12906-021-03238-w

**Published:** 2021-04-10

**Authors:** Fujiao Nie, Jiazhao Yan, Yanjun Ling, Zhengrong Liu, Chaojun Fu, Xiang Li, Yuhui Qin

**Affiliations:** 1 Hunan Provincial Key Laboratory for the Prevention and Treatment of Ophthalmology and Otolaryngology Diseases with Chinese Medicine, Hunan University of Chinese Medicine, Hanpu Rd., Yuelu District, Changsha, 410208 Hunan China; 2 Hunan Engineering Technological Research Center for the Prevention and Treatment of Otolaryngologic Disease and Protection of Visual Function with Chinese Medicine, Changsha, 410208 Hunan China; 3 Ophthalmology Department, The First Hospital of Hunan University of Chinese Medicine, Shaoshan Rd., Yuhua District, Changsha, 410007 Hunan China; 4 The Domestic First-class Discipline Construction Project of Chinese Medicine of Hunan University of Chinese Medicine, Hanpu Rd., Yuelu District, Changsha, 410208 Hunan China; 5 Institute of Chinese Medicine of Hunan Province, Lushan Rd., Yuelu District, Changsha, 410006 Hunan China

**Keywords:** Diabetic retinopathy, Apoptosis, Oxidative stress, Pericytes, Chinese herbal formula, Network pharmacology, PARP/GAPDH

## Abstract

**Background:**

Diabetic retinopathy (DR) has become a worldwide concern because of the rising prevalence rate of diabetes mellitus (DM). Despite much energy has been committed to DR research, it remains a difficulty for diabetic patients all over the world. Since apoptosis of retinal microvascular pericytes (RMPs) is the early characteristic of DR, this study aimed to reveal the mechanism of *Shuangdan Mingmu* (SDMM) capsule, a Chinese patent medicine, on oxidative stress-induced apoptosis of pericytes implicated with poly (ADP-ribose) polymerase (PARP) / glyceraldehyde 3-phosphate dehydrogenase (GAPDH) pathway.

**Methods:**

Network pharmacology approach was performed to predict biofunction of components of SDMM capsule dissolved in plasma on DR. Both PARP1 and GAPDH were found involved in the hub network of protein-protein interaction (PPI) of potential targets and were found to take part in many bioprocesses, including responding to the regulation of reactive oxygen species (ROS) metabolic process, apoptotic signaling pathway, and response to oxygen levels through enrichment analysis. Therefore, in vitro research was carried out to validate the prediction. Human RMPs cultured with media containing 0.5 mM hydrogen oxide (H_2_O_2_) for 4 h was performed as an oxidative-damage model. Different concentrations of SDMM capsule, PARP1 inhibitor, PARP1 activation, and GAPDH inhibitor were used to intervene the oxidative-damage model with N-Acetyl-L-cysteine (NAC) as a contrast. Flow cytometry was performed to determine the apoptosis rate of cells and the expression of ROS. Cell counting kit 8 (CCK8) was used to determine the activity of pericytes. Moreover, nitric oxide (NO) concentration of cells supernatant and expression of endothelial nitric oxide synthase (eNOS), superoxide dismutase (SOD), B cell lymphoma 2 (BCL2), vascular endothelial growth factor (VEGF), endothelin 1 (ET1), PARP1, and GAPDH were tested through RT-qPCR, western blot (WB), or immunocytochemistry (ICC).

**Results:**

Overproduction of ROS, high apoptotic rate, and attenuated activity of pericytes were observed after cells were incubated with media containing 0.5 mM H_2_O_2_. Moreover, downregulation of SOD, NO, BCL2, and GAPDH, and upregulation of VEGFA, ET1, and PARP1 were discovered after cells were exposed to 0.5 mM H_2_O_2_ in this study, which could be improved by PARP1 inhibitor and SDMM capsule in a dose-dependent way, whereas worsened by PARP1 activation and GAPDH inhibitor.

**Conclusions:**

SDMM capsule may attenuate oxidative stress-induced apoptosis of pericytes through downregulating PARP expression and upregulating GAPDH expression.

**Supplementary Information:**

The online version contains supplementary material available at 10.1186/s12906-021-03238-w.

## Background

With the improvement of people’s life and global population aging, the prevalence rate of diabetes mellitus (DM) has been rising and DM has become a major public concern, which results in multi-organ dysfunction including cardiovascular complications, diabetic nephropathy, peripheral neuropathy, and diabetic retinopathy (DR) [1]. DR is a chronic progressive sight-threatening complication of DM, mainly implicated with hyperglycemia, hyperlipidemia, hypertension, and anemia [2]. Much energy has been committed to revealing the pathogenesis of DR. Unfortunately, the specific mechanism remains vague to us. As so far, four main biological pathways have been discovered involved in the pathogenesis of hyperglycemic damage to microvascular cells: (1) Increased activation of polyol pathway [3]; (2) Upregulation of advanced glycation end products (AGEs) [3, 4]; (3) Overactivation of protein kinase C (PKC) [4]; (4) Upregulation of hexosamine pathway [3]. There is a “unified mechanism” of activation for these seemingly disparate biological pathways, which has been verified to be high glucose-induced mitochondria enhancing the overexpression of reactive oxygen species (ROS) and leads to the death of retinal microvascular cells ultimately [3]. Apoptosis of retinal microvascular pericyte cells (RMPs), embedded within the vascular basement membrane of the capillaries and essential in modulating microvascular physiology and pathological angiogenesis, plays a predominant role in the pathogenesis of early DR [5, 6]. However, the specific mechanism of ROS leading to loss of RMPs remains elucidated.

The effect of ROS is duplex and depends upon the quantity of generated ROS. A small rise of ROS can activate signaling pathways to initiate biological processes, called redox biology. Whereas, overexpression of ROS can bring damage to DNA and protein [7]. Oxidative stress (OS) is an imbalance between antioxidants and free radicals, eventually resulting in cell and tissue damage. Poly (ADP- ribose) polymerase (PARP), a family of enzymes implicated in many cellular processes, such as DNA repair, genomic stability, programmed cell death, and apoptosis, has been found to be overactivated in both ischemic and diabetic conditions [8].

Glyceraldehyde 3-phosphate dehydrogenase (GAPDH), an enzyme to catalyze the sixth step of glycolysis for energy and carbon molecules, besides participating in metabolic function, is found to be implicated in non-metabolic processes, such as initiation of apoptosis, axoplasmic transport, and transcription activation [9]. GAPDH has been found to initiate apoptosis involved in an activity mediated by GAPDH binding to DNA like in the transcription activation and participate in transcription of genes involved in antiapoptotic pathways and cell proliferation [10]. Moreover, GAPDH acts as a reversible metabolic switch under OS, involving oxidant-treatment-induced inactivation of GAPDH temporally re-route metabolic flux from glycolysis to the pentose phosphate pathway, allowing cells to generate more NADPH [11]. What’s more, GAPDH inhibition was found to be a consequence of poly (ADP ribosyl) ation of GAPDH by PARP, which was activated by DNA strand breaks produced by reactive species generated by hyperglycemia [12]. Both the hyperglycemia-induced decrease inactivation of GAPDH and its poly (ADP-ribosyl) ation can be prevented by overexpression of either uncoupling protein-1 or superoxide dismutase (SOD).

SDMM capsule (SDFA approval number: Z20080062), a Chinese patent medicine for DR and produced by Beijing Qihuang Pharmaceutical Co. LTD has been completed phase III trial and showed great potential in alleviating DM-induced symptoms, such as polyuria, polydipsia, polyphagia, and dysphoria with smothery sensation. Moreover, much work has been done to reveal the mechanism of SDMM capsule on DR and it was found to be capable of helping diabetic rats scavenged overproduction of ROS. In addition, components of SDMM capsule, which has been validated to be absorbed into plasma through the ultra-performance liquid chromatography quadrupole time of flight mass spectrometry (UPLC-Q-TOF·MS) analysis [13, 14], were performed network pharmacology analysis to predict potential targets of SDMM capsule on treating DR. And PARP1 and GAPDH were found to be among those potential targets as well as a densely connected network of protein-protein interaction (PPI) of potential targets. Moreover, enrichment analysis indicated those potential targets are mainly involved in *response to oxidative stress*, *regulation of reactive oxygen species metabolic process*, *apoptotic signaling pathway*, *response to oxygen levels*, and *regulation of cellular response to stress*. Given the critical role of apoptosis of RMPs in the early pathogenesis of DR, we preferred to study the effect of SDMM capsule on oxidative stress-induced apoptosis of pericytes implicated with PARP/GAPDH pathway, therefore.

## Methods

### Network pharmacology analysis was performed to predict potential targets of SDMM capsule on treating DR

Network pharmacology analysis was performed to predict potential targets of ingredients of SDMM capsule indigested into plasma on treating DR. Our group has figured out components of SDMM capsule absorbed into plasma by analyzing the blood of SD rats gavaged with SDMM capsule through UPLC-Q-TOF·MS technology in previous work. Thirty-six kinds of components including three kinds of metabolites, glucuronic acid conjugates of protocatechuic acid, glycine conjugates of Danshensu (Propanoic acid, 3-(3,4-dihydroxy phenyl)-2-hydroxy-), and D-ring-opening metabolites of crypto tanshinone, were identified [13].

#### Collecting DR pathogenic genes and ingredients targets of SDMM capsule

DR pathogenic genes were acquired from DisGeNET, Genecards, OMIM, and Drugbank database. And genes names were corrected at the Uniprot database. Meanwhile, ingredients of SDMM capsule absorbed into plasma were collected to accomplish targets predictions through the Swiss Target Prediction database. Eventually, we got intersection genes between ingredients targets and DR pathogenic genes, namely potential targets of SDMM capsule on treating DR.

#### Setting up PPI network

Potential targets were imported into the String database to analyze protein-protein interaction (PPI) of those potential targets. Medium confidence of 0.4 was set to ensure the reliability of data. String database output a network and an Excel sheet of analysis results. To set up a beautiful PPI network, the Excel sheet was put into Cytoscape 3.7.2 software. Moreover, the color and size of the network nodes were set according to Degree value. That is to say, the darker and larger the nodes, the more important the nodes may be among the network.

#### Visualize and analyze biomolecular relationships of potential targets

BisoGenet plugin of Cytoscape 3.7.2 was used to visualize and analyze biomolecular relationships of those potential targets. Ingredients targets and DR disease targets were respectively accomplished BisoGenet analysis. Subsequently, the intersection of the two networks was merged and interaction data were analyzed with the CytoNCA plugin. Subsequently, analyzed data, such as Degree value, Betweenness, Closeness, LAC, Neighborhood Connectivity, and Stress, was applied to extract hub network.

#### Enrichment analysis

Kyoto Encyclopedia of Genes and Genomes (KEGG) pathway and gene ontology (GO) enrichment analysis were performed at the Metascape database to annotate the function of potential targets. Human Species was set; minimum overlap was set at 3; minimum enrichment value was set at 0.5; and *P*<0.01. Moreover, Molecular Complex Detection (MCODE) algorithm was used to identify densely connected networks.

### In vitro research

#### Cells

Human retinal microvascular pericytes (HRMVPs) (Zhongqiao Xinzhou Biological Company, ZQ0878) were cultured in media supplemented with 10% fetal bovine serum (FBS) (Gibco 04–001-1ACS) and 1% Dulbecco’s modified eagle medium (DMEM) (Sigma D5796) containing two antibodies (Beyotime, SV30010) in a humidified incubator with 5% CO_2_ at 37 °C. HRMVPs were randomly divided into ten groups, *Blank*, *Control*, *NAC* (positive control group), *High SDMM* (high concentration of SDMM capsule), *Medium SDMM* (medium concentration of SDMM capsule), *Low SDMM* (low concentration of SDMM capsule), *PARP -* (PARP inhibitor), *PARP +* (PARP activation), *GAPDH -* (GAPDH inhibitor), and *H SDMM/PARP +* (high concentration of SDMM capsule plus PARP activation) group.

#### SDMM capsule concentration sifting

Concentrations of SDMM capsule (Beijing Qhuang Pharmaceutical Co. LTD, Z20080062) were set as follow: 0 mg/ml, 0.078 mg/ml, 0.156 mg/ml, 0.313 mg/ml, 0.625 mg/ml, 1.25 mg/ml, 2.5 mg/ml, 5 mg/ml, and 10 mg/ml. Logarithmic growth phase cells were seeded in 96 well plates with 5*10^3^ cells and 100 μL media per well and each concentration occupies six wells. After 24-h incubation, media containing different concentrations of SDMM capsule was removed and 10 μL cell counting kit 8 (CCK8) (DOJINDO, NU679) solution dissolved in DMEM was added into every well to determine the activity of pericytes. After four-hour incubation, the optical density (OD) value was analyzed by a Bio-tek microplate reader at 450 nm wavelength.

#### Intervening measures

After concentration sifting, maximum non-toxic concentration (TC0) of SDMM capsule incubating HRMVPs was acquired. Subsequently, cells are divided into ten groups as mentioned above, and intervened with different reagents. Cells in *Blank* group were cultured in media without drug; cells in *Control* group were incubated with media containing 0.5 mM hydrogen peroxide (H_2_O_2_) for 4 h; cells in *NAC*, *High SDMM*, *Medium SDMM*, *Low SDMM*, *PARP-*, *PARP+*, *GAPDH-*, and *H SDMM/PARP+* group were all primarily incubated with 0.5 mM H_2_O_2_ for 4 h, subsequently, intervened with different agents as follow: cells in *NAC* group was cultured with media containing 0.5 mM N-Acetyl-L-cysteine (NAC) for 24 h; cells in *High SDMM* group were cultured with media containing TC0 SDMM capsule for 24 h; cells in *Medium SDMM* group were followed with media containing 1/2 TC0 of SDMM capsule for 24 h; cells in *Low SDMM* group were cultured with media containing 1/4 TC0 of SDMM capsule for 24 h; cells in *PARP -* group were cultured in media containing 5 mg/ml PARP inhibitor (APExBIO, A4154) for 24 h; cells in *PARP +* group were cultured in media containing 3 nM PARP activation (MCE, 1639792–20-3) for 24 h; cells in *GAPDH -* group were cultured in media containing 80 uM GAPDH inhibitor (APExBIO, C4003) for 24 h; cells in *H SDMM/PARP +* group were incubated with media containing TC0 SDMM capsule plus 3 nM PARP activation for 24 h.

#### Apoptosis

Flow cytometry technology was performed to detect the apoptosis rate of HRMVPs. Cells were digested with 0.25% pancreatic enzymes containing 0.02% EDTA and went through two times centrifugation, which was followed with PBS wash. Subsequently, 500 μL binding buffer, 5 μL Annexin V-FITC (Nanjing Kaiji, KGA108), and 5 μL propidium iodide (PI) were added into cells successively. Then, the cell suspension was incubated out of light at room temperature for 15 min. Immediately, the green fluorescence of annexin V-FITC with excitation wavelength (Ex) at 488 nm and emission wavelength (Em) at 530 nm was detected in the FITC channel (FL1) and the red fluorescence of PI with Ex at 488 nm and Em ≥ 630 nm was determined in FL2 (PE) channel. Phosphatidylserine (PS) is only distributed on the inner side of the lipid bilayer of the cell membrane. However, PS in the cell membrane turns from inside to outside of the lipid membrane in the early stage of apoptosis. And Annexin V, a kind of Ca^2+^-dependent phospholipid-binding protein with a molecular weight of 35–36 KD, has a high affinity with PS. Thus, Annexin V labeled with FITC can act as one of the sensitive indexes to detect early apoptosis. Whereas, propidium iodide (PI), a kind of nucleic acid dye, can’t pass through the normal cell membrane, but it can go through the cell membrane of the middle-late stage apoptotic and dead cells to dye the nucleus red. Therefore, Annexin V in conjunction with PI can distinguish cells in different apoptotic stages.

#### NO concentration

As NO is an active signal molecule and is quickly converted into NO_3_^−^ in vivo, the specificity of nitrate reductase was used to reduce NO_3_^−^ to NO_2_^−^ and the concentration was measured through color depth. Before detecting NO concentration, cells were cultured in medium without serum for 24 h. Then, cellular supernatant was collected to detect the NO concentration. The specific procedures of determining NO concentration followed the instruction of the NO kit (Nanjing Jiancheng, A012–1).

#### Fluorescence intensity of ROS

Flow cytometry was performed to determine the fluorescence intensity of ROS. Firstly, cells were digested with 0.25% trypsin containing 0.02% EDTA. Subsequently, the supernatant was removed after centrifugation, and cells were washed with media and centrifuged once again. Secondly, 1:1000 2′,7′-Dichlorodihydrofluorescein diacetate (DCFH-DA) solution dissolved by DMEM was added into cells. Then, the cell suspension was incubated out of light at 37 °C for 20 min and mixed every 3–5 min. Finally, the fluorescence intensity of ROS was tested in the FITC channel at Ex/Em = 488/530 nm after cells were washed with DMEM two times.

#### mRNA expression

RT-qPCR was performed to determine mRNA expression of nitric oxide synthase (NOS), SOD, endothelial-1(ET1), VEGF, BCL-2, PARP1, and GAPDH. Primarily, total RNA was extracted using Trizol (America Thermo, 15,596,026) following the manufacturer’s instructions. Then, a reverse transcription kit (Kangwei century, CW2569, CW2141) was adopted to accomplish reverse transcription. SYBR method was used to complete qPCR. Search the sequence of the target gene on the National Center for Biotechnology Information (NCBI). And took advantage of primer 5 software to design primer. All mRNA expression was expressed as a ratio to actin.

#### Protein expression

Western blotting (WB) was performed to determine protein expression of eNOS, ET1, VEGFA, BCL-2, PARP1, and GAPDH. Firstly, the protein was extracted with RIPA buffer (Beyotime Biotechnology, P0013B), followed by ultrasonication and centrifugation. Subsequently, the concentration of protein was measured with a BCA assay kit. Then, western blotting was performed. The primary antibodies were set as follow: 1:5000 β-actin (Mouse; America Proteintech, 66,009–1-Ig), 1:50000 SOD (Rabbit; Abcam, ab51254), 1:1000 ET1 (Mouse; Abcam, ab2786), 5 μg/mL VEFGA (Mouse; Abcam, ab1316), 1:1000 BCL2 (Rabbit; Abcam, ab32124), and 1:2000 PARP1 (Rabbit; Abcam, ab32138). The primary antibodies were incubated at room temperature for 90 min. Then, it came to the second antibody incubation. The second antibodies were HRP goat anti-mouse IgG (America Proteintech, SA00001–1) at 1:5000 dilution ratio and HRP goat anti-rabbit IgG (America Proteintech, SA00001–2) at 1:6000 dilution ratios. At last, an enhanced chemiluminescence (ECL) reaction was performed to visualize antibodies.

#### Immunocytochemistry

Immunocytochemistry (ICC) technique was performed to anatomically visualize the localization and expression of VEGF and PARP in HRMVPC. HRMVPC growing on glass coverslips were fixed with 4% paraformaldehyde for 30 min, then, incubated with 0.3% Triton at 37 °C for 30 min and 3% H_2_O_2_ was added to inactivate endogenous enzymes. Subsequently, the primary antibodies were added and incubated overnight at 4 °C. Then, the second antibodies were incubated at 37 °C for 30 min. All the above procedures were followed with a PBS wash. Then, the DAB kit was performed as instruction, and slips were washed with distilled water. Subsequently, hematoxylin was used to counterstain for 5–10 min and followed with distilled water and PBS wash. Eventually, dehydration was performed with ethanol, and fixation was accomplished. The observation was under a microscope and images were captured. Eventually, the ICC-analysis plugin of Image J software was used to quantify protein expression.

## Statistical analysis

Data were presented as mean ± SD. For the indicated number of independently performed experiments using the SPSS package (SPSS 24.0 for windows). The statistical significance within parameters was evaluated by one-way analysis of variation (ANOVA), followed by post hoc test with the least significant differences (LSD). Significant differences were classified as: * for *P*<0.05, ** *P*<0.01.

## Results

### Network pharmacology analysis

#### PARP1 and GAPDH were predicted to be potential targets of SDMM capsule on treating DR

**Fig. 1:**
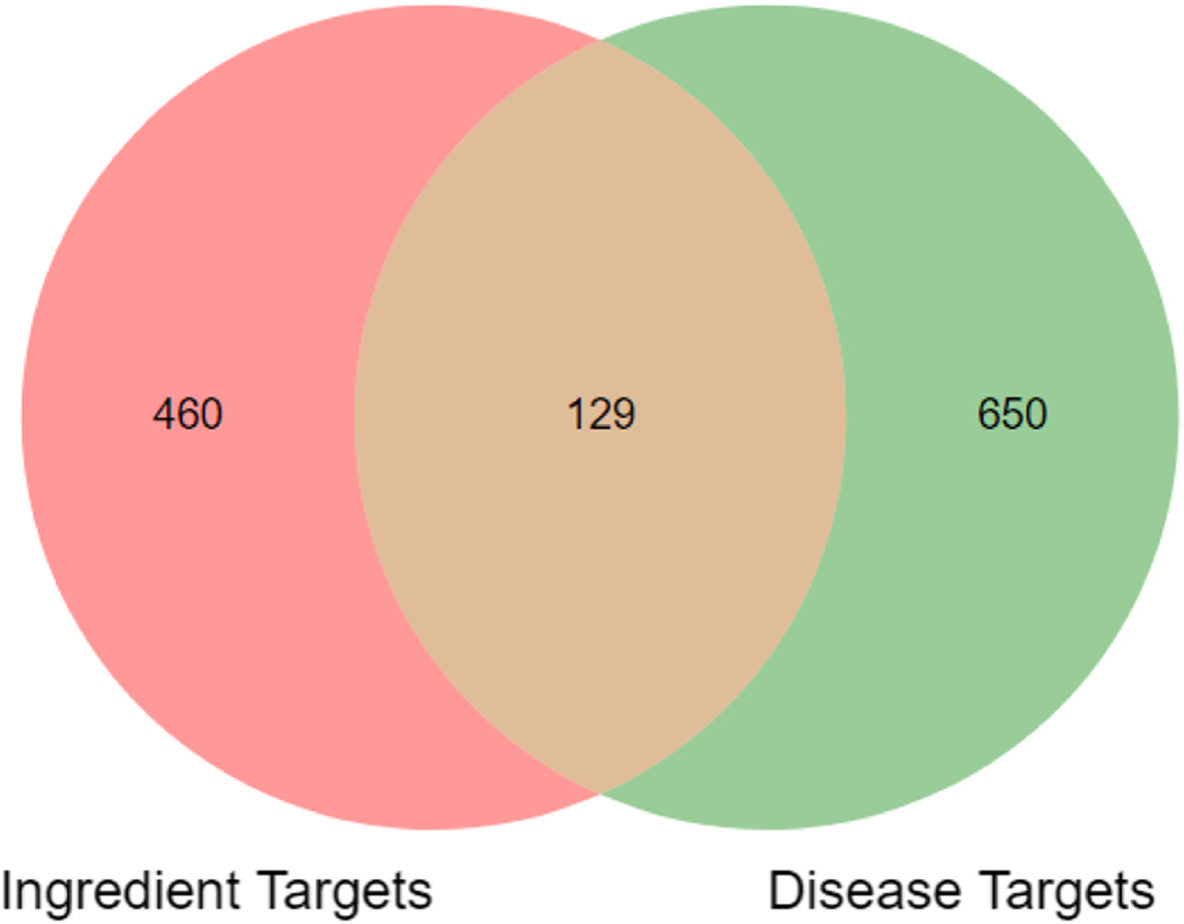
Venn chart. 589 kinds of ingredients targets of SDMM capsule were predicted through the SWISS Target Prediction database. And 779 kinds of DR disease targets were achieved at DisGeNET, Genecards, OMIM, and Drugbank database. Afterwards, 129 intersection gene, namely potential targets, were acquired

**Fig. 2:**
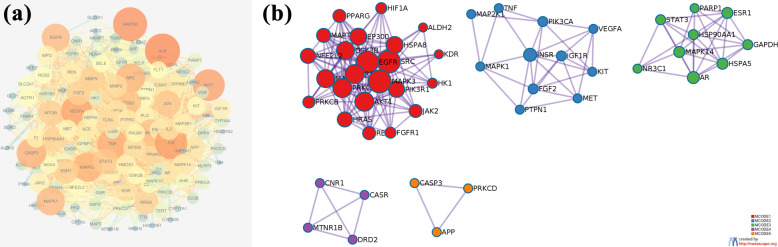
Protein-protein interaction (PPI) network. **a** A 129-node and 2167-edge PPI network of potential targets was acquired at the String database. The size of nodes was set according to Degree value. Namely, the higher the Degree value the bigger the image. **b** Molecular Complex Detection (MCODE) algorithm was applied to identify the densely connected network components. Five powerful functional networks were identified. Notably, PARP1, GAPDH, and VEGFA are all among those densely connected networks

Table 1 shows the molecular information of SDMM capsule components absorbed into plasma. Five hundred eighty-nine kinds of components targets were predicted at SWISS Target Prediction software. And 779 kinds of the pathogenic gene of DR were collected from the database mentioned above. Therefore, a VENN diagram was got and 129 intersection targets, namely potential targets of SDMM capsule on treating DR were acquired, including PARP1, GAPDH, VEGFA, SOD, and NOS (Fig. 1) [15, 16]. Then, potential targets were analyzed at String database and we got the analysis result of the protein-protein interaction (PPI) network (Fig. 2a).

**Table 1:** Molecular information of ingredients absorbed into plasma of SDMM capsule

NO.	Compound	Molecular formula	Source
1	L-Tyrosine tert-butyl ester	C_13_H_19_NO_3_	*Cornus officinalis* Sieb. et Zucc.
2	Sarracenin	C_11_H_14_O_5_	*Cornus officinalis* Sieb. et Zucc.
3	Sugiol	C_20_H_28_O_2_	*Salvia miltiorrhiza* Bunge
4	Cryptotanshinone	C_19_H_20_O_3_	*Salvia miltiorrhiza* Bunge
5	3,4-Dihydroxybenzoic acid	C_7_H_6_O_4_	*Alisma plantago-aquatica* L.
6	N-Acetyl-L-proline	C_7_H_11_NO_3_	*Cornus officinalis* Sieb. et Zucc.
7	2-cyclohexylethyl-beta-D-maltoside	C_20_H_36_O_11_	*Eclipta prostrata* (L.) L.
8	Paeonilactone B	C_10_H_12_O_4_	*Paeonia suffruticosa* Andr.
9	Apigenin	C_15_H_10_O_5_	*Eclipta prostrata* (L.) L.
10^a^	D-ring-opening metabolites of cryptotanshinone	C_19_H_22_O_4_	*Salvia miltiorrhiza* Bunge
11	5-Allyl-1,3-bis (methoxymethyl)-5-(1-methylbutyl) barbituric acid	C_16_H_26_N_2_O_5_	*Dioscorea polystachya* Turczaninow
12	Loliolide	C_11_H_16_O_3_	*Eclipta prostrata* (L.) L. *(mohanlian,*
13	Pachymic acid C	C_31_H_46_O_4_	*Poria cocos* (Schw.) Wolf.
14	Loganin	C_17_H_26_O_10_	*Cornus officinalis* Sieb. et Zucc.
15	Coniferylaldehyde	C_10_H_10_O_3_	*Eclipta prostrata* (L.) L.
16	Loganetin	C_11_H_16_O_5_	*Cornus officinalis* Sieb. et Zucc.
17	Catalpin	C_13_H_20_O_9_	*Eclipta prostrata* (L.) L*.*
18	Eriodictyol	C_15_H_12_O_6_	*Eclipta prostrata* (L.) L.
19	Albiflorin	C_23_H_28_O_11_	*Paeonia suffruticosa* Andr.
20	Cosmosiin	C_21_H_20_O_10_	*Ligustrum lucidum* Ait.
21	Resveratrol	C_14_H_12_O_3_	*Smilax glabra* Roxb.
22	Oriediterpenoside	C_25_H_40_O_6_	*Alisma plantago-aquatica* L.
23	7-O-Methyl morroniside	C_18_H_28_O_11_	*Cornus officinalis* Sieb. et Zucc.
24	oleanolic acid acetate	C_32_H_50_O_4_	*Achyranthes bidentata* Blume
25	Cornuside	C_24_H_30_O_14_	*Cornus officinalis* Sieb. et Zucc.
26	Oleanic acid	C_30_H_48_O_3_	*Eclipta prostrata* (L.) L.
27^a^	Glucuronic acid conjugates of protocatechol	C_6_H_10_O_7_	*Alisma plantago-aquatica* L.
28^a^	Glycine conjugates of Danshensu	C_9_H_10_O_5_	*Salvia miltiorrhiza* Bunge
29	Nuezhengalaside	C_18_H_28_O_9_	*Eclipta prostrata* (L.) L.
30	Ecliptin	C_36_H_58_O_8_	*Eclipta prostrata* (L.) L.
31	Acetylursolic acid	C_32_H_52_O_4_	*Poria cocos (Schw.)* Wolf.
32	Skullcapflavone II	C_19_H_18_O_8_	*Eclipta prostrata* (L.) L.
33	Alisol A	C_30_H_50_O_5_	*Alisma plantago-aquatica* L.
34	Luteolin	C_15_H_10_O_6_	*Eclipta prostrata* (L.) L.
35	Isocryptotanshinone	C_19_H_20_O_3_	*Salvia miltiorrhiza* Bunge
36	Tanshinone IIA	C_19_H_18_O_3_	*Salvia miltiorrhiza* Bunge

(^a^: Metabolites)

**Fig. 3:**
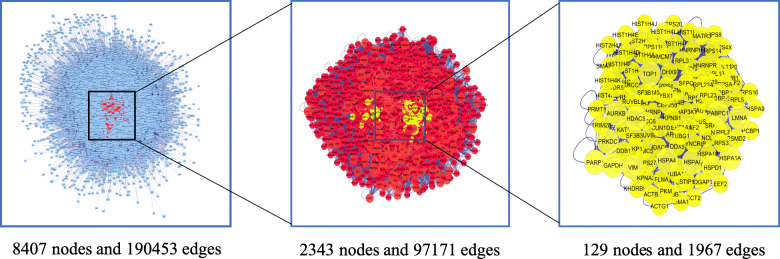
Visualization and analysis of biomolecular relationships of potential targets. BisoGenet plugin of Cytoscape 3.7.2 was applied to visualize and analyze biomolecular relationships of intersection gene. Hithubs network, the red one, was acquired through extracting nodes whose Degree value were above two-fold median (81*2 = 162). Afterwards, median of Betweenness, Closeness, Degree, LAC, Neighborhood Connectivity, Stress, and Radiality were used to extract a hub network with 129 nodes and 1967 edges, the yellow one. Of notice, PARP1 and GAPDH were both involved in the yellow hub network

Taking advantage of the visualized and analyzed function of the BisoGenet plugin of Cytoscape 3.7.2, we got biomolecular relationships of those potential targets, and hub network was extracted through Degree, Betweenness, Closeness, LAC, Neighborhood Connectivity, Stress, and Radiality value (Fig. 3). Moreover, both PARP1 and GAPDH are implicated in the hub network, indicating the powerful potential of PARP1 and GAPDH participating in the mechanism of SDMM capsule treating DR.

#### Enrichment analysis

**Fig. 4:**
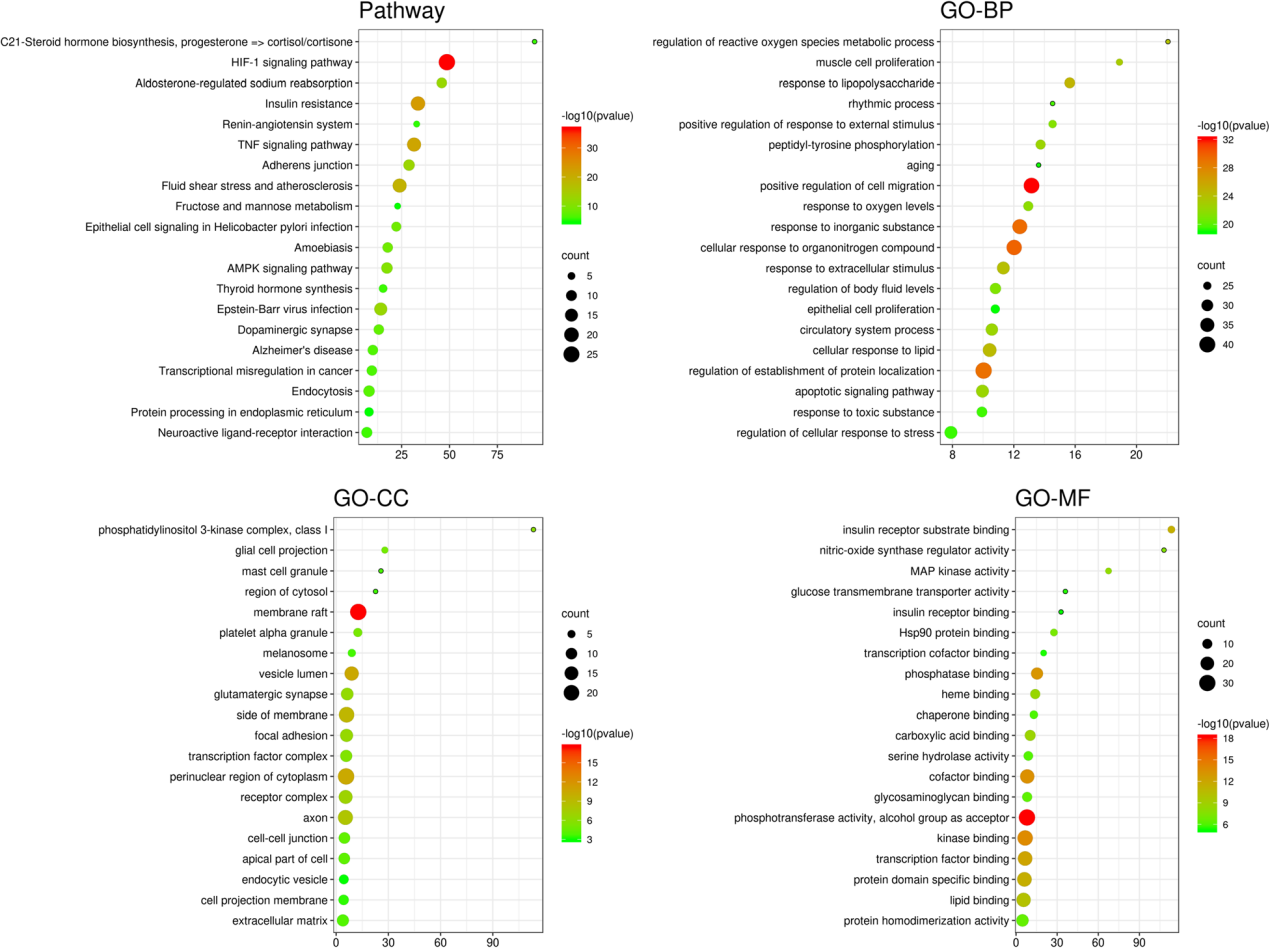
Enrichment analysis. Top 20 ontology items are displayed in the enrichment images. *Count* represents number of gene involved in every item and the bigger the -log 10 (*p* value), the more important the item. It’s clearly displayed at the diagram that KEGG pathway includes HIF-1 signaling pathway, insulin resistance, TNF signaling pathway, and AMPK signaling pathway. What attracted our eyes are items figured out through GO-BP analysis including regulation of reactive oxygen species metabolic process, apoptotic signaling pathway, response to oxygen levels, regulation of cellular response to stress, response to toxic substance, aging, and cellular response to organonitrogen compound, and items analyzed through GO-MF, such as nitric-oxide synthase regulator activity, insulin receptor binding, glucose transmembrane transporter activity, insulin receptor substrate binding, phosphotransferase activity, alcohol group as acceptor, and kinase binding

Enrichment analysis of potential targets was carried out at the Metascape database with *Min Overlap* set as 3, *P value cutoff* setting as 0.01, and *minimum Enrichment* set as 1.5. Finally, we got 2254 ontology items (*P* < 0.01), among which GO-BP ontology with 2013 entries are the most, mainly related to *regulation of reactive oxygen species metabolic process*, *apoptotic signaling pathway*, *response to oxygen levels*, *regulation of cellular response to stress*, *response to oxidative stress*, *response to toxic substance*, *aging*, and *cellular response to organonitrogen compound*; 88 entries are related to GO-CC, including *phosphatidylinositol 3-kinase complex*, *membrane raft*, *vesicle lumen*, *perinuclear region of cytoplasm*, *glial cell projection*, *cell-cell junction*, *extracellular matrix*, *apical part of cell*, *organelle outer membrane*, *cell body*, *outer membrane*, *lytic vacuole*, *lysosome*, *apical plasma membrane*, and *endocytic vesicle*; And there are 153 items related to GO-MF, mainly involving *nitric-oxide synthase regulator activity*, *insulin receptor binding*, *glucose transmembrane transporter activity*, *insulin receptor substrate binding*, *phosphotransferase activity*, *alcohol group as acceptor*, *kinase binding*, *protein tyrosine kinase activity*, *protein serine/threonine kinase activity*, *transmembrane receptor protein tyrosine kinase activity*, *growth factor binding*, *cofactor binding*, *oxidoreductase activity acting on the CH-OH group of donors*, *NADP binding*, *phosphatase binding*, and *oxygen binding* (Fig. 4).

What’s more, five densely connected network components were figured out through enrichment analysis, indicating a powerful functional group (Fig. 2b). Furthermore, PARP1 and GAPDH are involved in the densely connected network components, related to *response to oxidative stress*, *regulation of cellular amide metabolic process*, *regulation of insulin secretion*, *positive regulation of kinase activity*, *phosphotransferase activity*, *protein tyrosine kinase activity*, and *enzyme activator activity*.

All the above predicted the strong potential of SDMM capsule attenuating OS-induced apoptosis of through the PARP/GAPDH pathway. Therefore, in vitro research was performed to verify the prediction.

### In vitro research

#### Concentration sifting of SDMM capsule

**Fig. 5:**
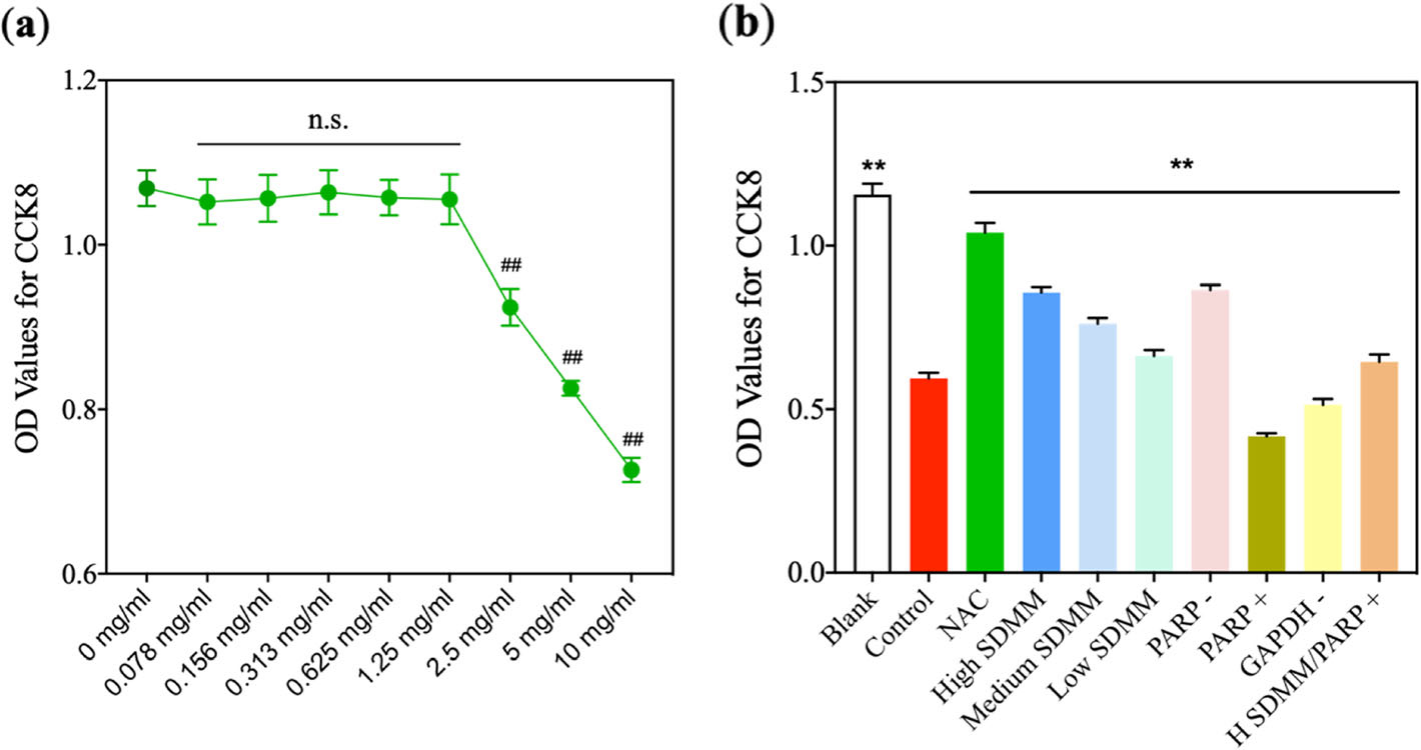
The activity of HRMVPs was detected by CCK8 kit (*n* = 6). **a** displays the activity of cells cultured with different concentrations of SDMM capsule. The activities of cells cultured with concentrations above 2.5 mg/ml were significantly lower than that of 0 mg/ml (*P*<0.01). **b** shows the activity of HRMVPC cultured with different agents. The activity of *Control* group was obviously lower than those of *Blank*, *NAC*, *High SDMM*, *Medium SDMM*, *Low SDMM*, *PARP -*, and *High SDMM/PARP +* group and statistically higher than that of *PARP +* and *GAPDH -* group (*P*<0.01). (##: versus 0 mg/ml, *P*<0.01; **: versus *Control* group, *P*<0.01)

After 24 h incubation in different concentrations of SDMM capsule, the activity of cells incubated with 0.078 mg/ml, 0.156 mg/ml, 0.313 mg/ml, 0.625 mg/ml, and 1.25 mg/ml SDMM capsule has no statistical difference compared with that of 0 mg/ml (*P*>0.05). Whereas, the activity of cells cultured with concentrations above 1.25 mg/ml was statistically lower than 0 mg/ml (*P*<0.01), indicating 1.25 mg/ml is the TC0 of SDMM capsule in culturing HRMVPs. (Fig. 5a).

#### Oxidative stress induced low activity of HRMVPs

CCK8 was performed to detect the activity of HRMVPs. After being incubated in 0.5 mM H_2_O_2_ for 4 h, the activity of HRMVPC was extraordinarily attenuated, which was improved by NAC, SDMM capsule, and PARP inhibitor and was inversely aggravated after being incubated with PARP activation and GAPDH inhibitor (Fig. 5b). Of notice, the activity of cells cultured with high concentration SDMM capsule was higher than the medium and low concentration of SDMM capsule, namely, SDMM capsule may concentration-dependently save cells’ activity.

#### Overproduction of ROS

**Fig. 6:**
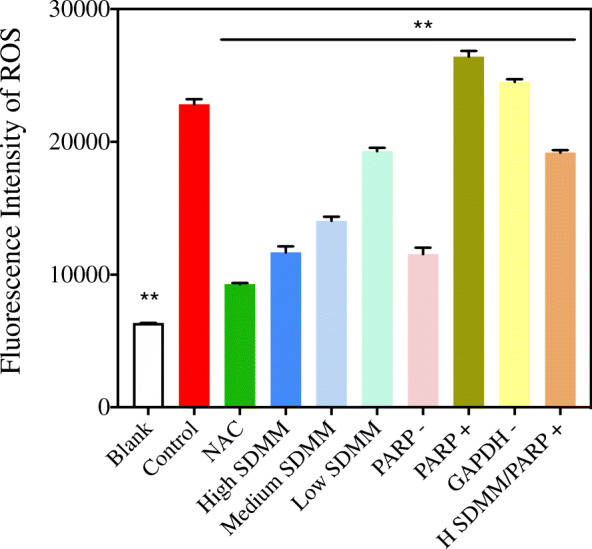
Fluorescence intensity of ROS (*n* = 3). The fluorescence intensity of ROS of *Control* group was obviously higher than *Blank*, *NAC*, *High SDMM*, *Medium SDMM*, *Low SDMM*, *PARP -*, and *High SDMM/PARP +* group, however, statistically lower than that of *PARP +* and *GAPDH +* group (*P*<0.01). (**: versus *Blank* group, *P*<0.01)

Flow cytometry was performed to detect the production of ROS (Fig. 6). As shown in Fig. 6, the fluorescence intensity of ROS of cells cultured with 0.5 mM H_2_O_2_ is significantly higher than the blank group. Whereas, SDMM capsule and PARP inhibitor can attenuate the overproduction of ROS. On the contrary, PARP activation and GAPDH inhibitor exacerbated the overexpression of ROS.

#### Oxidative stress-induced downregulation of NO

**Fig. 7:**
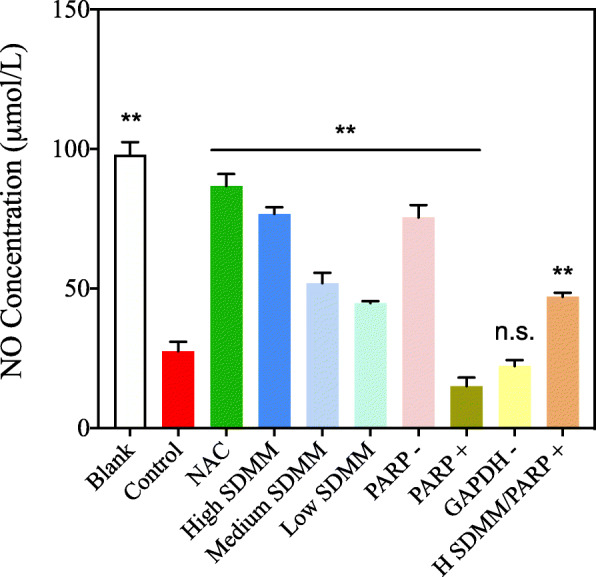
Concentration of NO (*n* = 3). NO concentration of *Control* group was statistically lower than *Blank*, *NAC*, *High SDMM*, *Medium SDMM*, *Low SDMM*, *PARP -*, and, *High SDMM/PARP +* group, however, obviously higher than *PARP +* group (*P*<0.01). (**: versus group B, *P*<0.01; n.s.: versus group B, *P*>0.05)

Nitric oxide (NO) is a pivotal signaling molecule involved in many physiological and pathological processes, which can be produced by NOS. As NO is an active signal molecule and is quickly converted into NO_3_^−^ in vivo, the specificity of nitrate reductase was used to transfer NO_3_^−^ to NO_2_^−^ and the concentration was measured through color depth (Fig. 7). NO concentration of the *Control* group is distinctly lower than the *Blank* group, indicating the injury resulting from OS can lead to the downregulation of NO. Whereas, NAC, SDMM capsule, and PARP inhibitor can upregulate the concentration of NO. Conversely, PARP activation can exacerbate the OS-induced downregulation of NO. What’s more, the concentration of NO of *H SDMM/PARP +* group is statistically higher than *Control* group (*P*<0.01), demonstrating the high-concentration SDMM capsule may attenuate the side effect resulting from PARP activation.

**Fig. 8:**
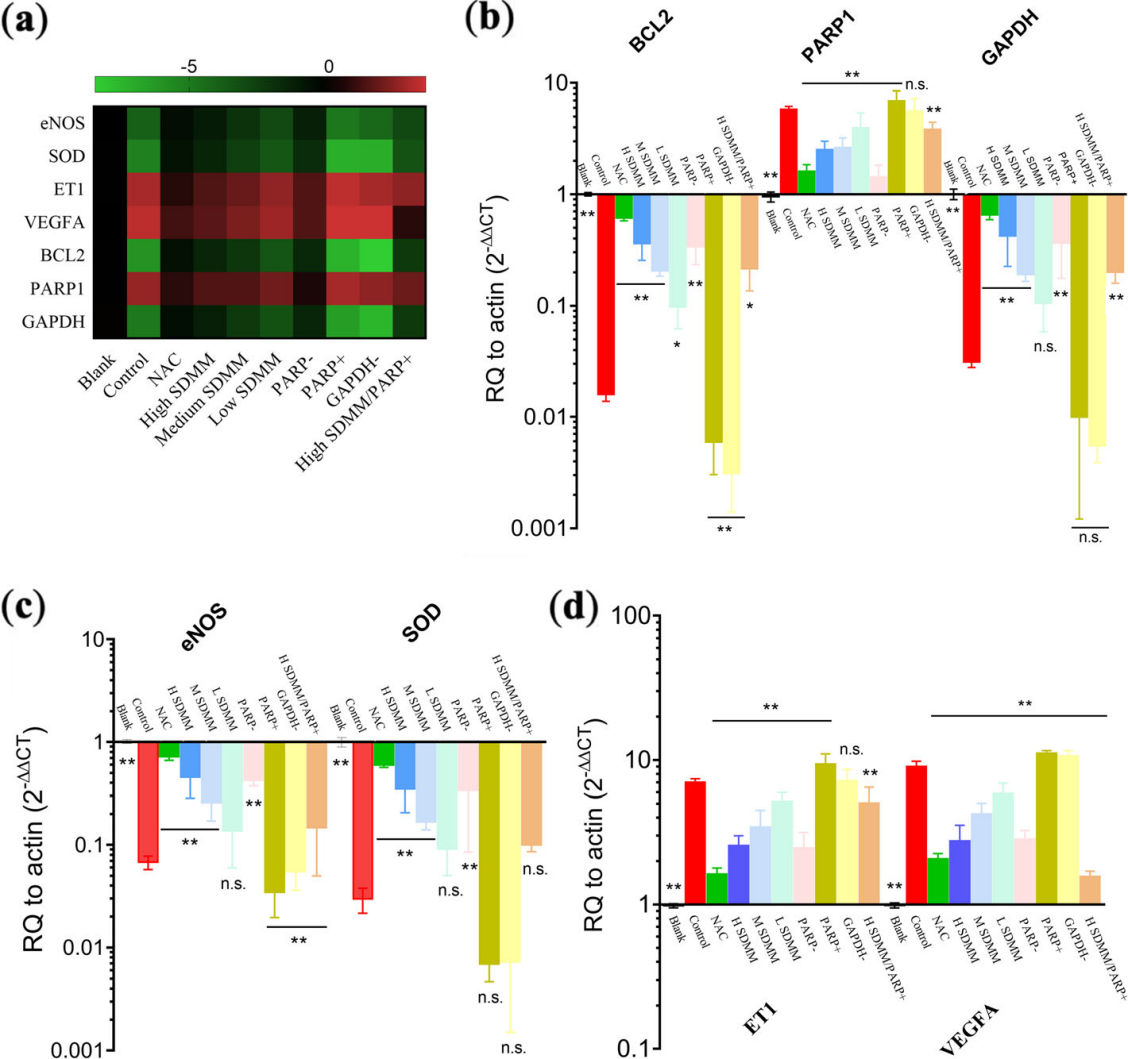
RT-qPCR (*n* = 3). RT-qPCR was performed to determine mRNA expression of eNOS, SOD, ET1, VEGFA, BCL2, PARP1, and GAPDH. **a** is the heatmap (−△△CT) and (**b**,**c**,**d**) display the fold change (2-△△CT) of those gene. It’s clearly shown that eNOS, SOD, BCL2, and GAPDH were downregulated in *Control* group, whereas, ET1, VEGFA, and PARP1 were upregulated in *Control* group. SDMM showed dose-dependent ability to resist above regulations. (**: versus *Control* group, *P*<0.01; *: versus *Control* group, *P*<0.05; n.s.:versus *Control* group, *P*>0.05)

As we all know, there are three kinds of known isoforms of NOS, namely neuronal nitric oxide synthase (nNOS), endothelial nitric oxide synthase (eNOS), and inducible nitric oxide synthase (iNOS) [17]. Since NO formed by eNOS takes part in ocular hemodynamic abnormalities that seem to trigger generation and progression of various ocular diseases including ischemic retinopathies for instance DM, the mRNA and protein expression of eNOS was respectively measured to confirm the regulating tendency of eNOS [18]. Both mRNA and protein expression of the *Control* group is sharply lower than the *Blank* group, suggesting eNOS could be expressed in HRMVPs and oxidative damage may bring about downregulation of eNOS, thus lead to decreased NO. Whereas, oxidative damage-induced attenuated eNOS can be improved by NAC, high concentration of SDMM capsule, and PARP inhibitor. However, eNOS expression of cells in the PARP activation group is lower than the *Control* group but the comparison shows no statistical difference (*P*>0.05) (Fig. 8).

#### Oxidative stress resulted in downregulation of SOD

RT-qPCR and ICC were performed to respectively detect mRNA and protein expression of SOD. As shown in Fig. 8a and c, the mRNA expression of SOD was extremely downregulated in *Control*, *PARP +*, and *GAPDH -* group, which was significantly lower than cells treated with NAC, high SDMM, medium SDMM, and PARP inhibitor (*P*<0.01). As we all know, SOD is an important antioxidant enzyme, characteristic of scavenging free radical and antioxidant capacity. Therefore, we concluded that oxidative damage can attenuate the antioxidant capacity of HRMVPs, which could be improved by PARP inhibitor and SDMM capsule in a concentration-dependent manner.

#### Oxidative stress stimulated HRMVPs to upregulate VEGF and ET1

**Fig. 9:**
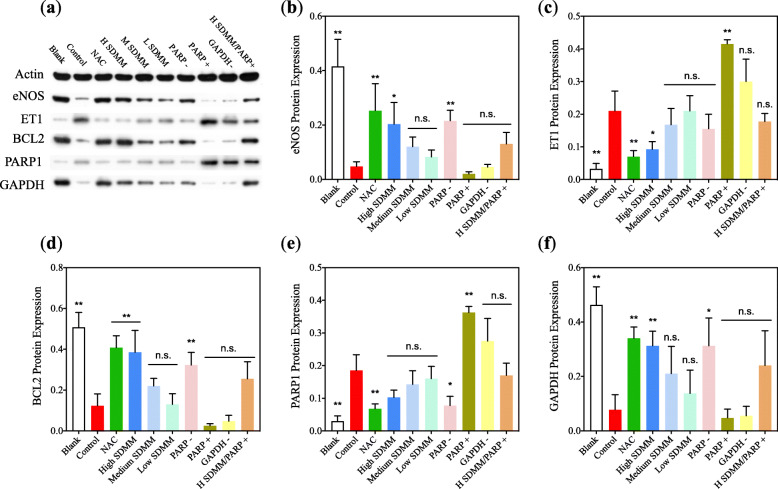
Western Blot (WB) (*n* = 3). WB was conducted to detect the protein expression of eNOS, ET1, BCL2, PARP1, and GAPDH. Data was expressed as a ratio to actin. The WB results were in accordance with RT-qPCR. (**: versus *Control* group, *P*<0.01; *: versus *Control* group, *P*<0.05; n.s.: versus *Control* group, *P*>0.05)

**Fig. 10:**
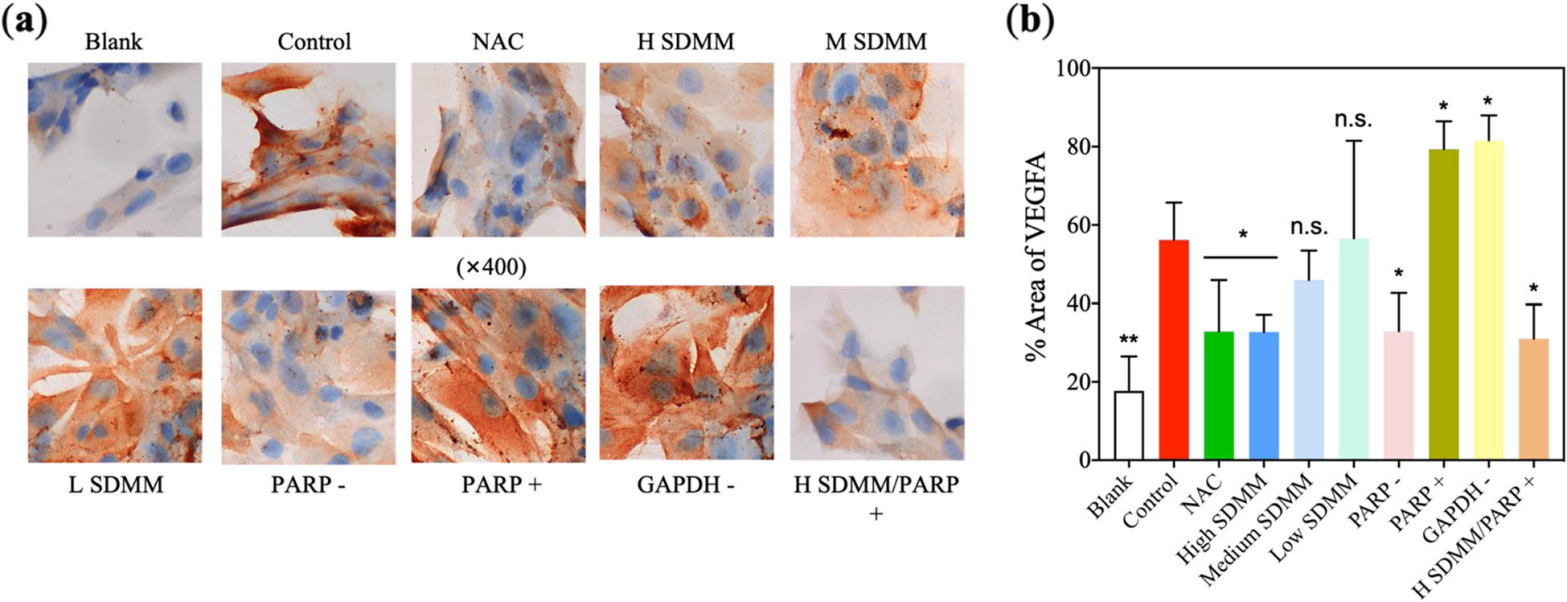
ICC of VEGFA. **a** shows representative ICC images. And ICC-analysis plugin of ImageJ was applied to quantify VEGFA protein expression, shown in (**b**) (*n* = 3). VEGFA expression of *Control* group is obviously higher than *Blank* group (*P*<0.01), and statistically higher than *NAC*, *H SDMM*, *PARP -*, and *H SDMM/PARP +* group (*P*<0.05). Whereas, the VEGFA expression of *Control* group was lower than *PARP +* and *GAPDH +* group (*P*<0.05). (**: versus *Control*, *P*<0.01; *: versus *Control*, *P*<0.05; n.s.: versus *Control*, *P*>0.05)

Vascular endothelial growth factor (VEGF) and endothelial 1 (ET1) is originally known as an essential growth factor secreted by endothelial cells [19]. Additionally, VEGF has been found to be expressed in non-endothelial cells. In this study, VEGF was verified to be expressed at an extremely low dose by HRMVPs. And both VEGF and ET1 were found to be upregulated by H_2_O_2_-incubated HRMVPs, which could be improved by the high concentration of SDMM capsule and PARP inhibitor, however, worsened by PARP1 activation and GAPDH inhibitor (Fig. 8a, d, Fig. 9c, and Fig. 10).

#### Oxidative stress induced increased apoptosis to HRMVPs

**Fig. 11:**
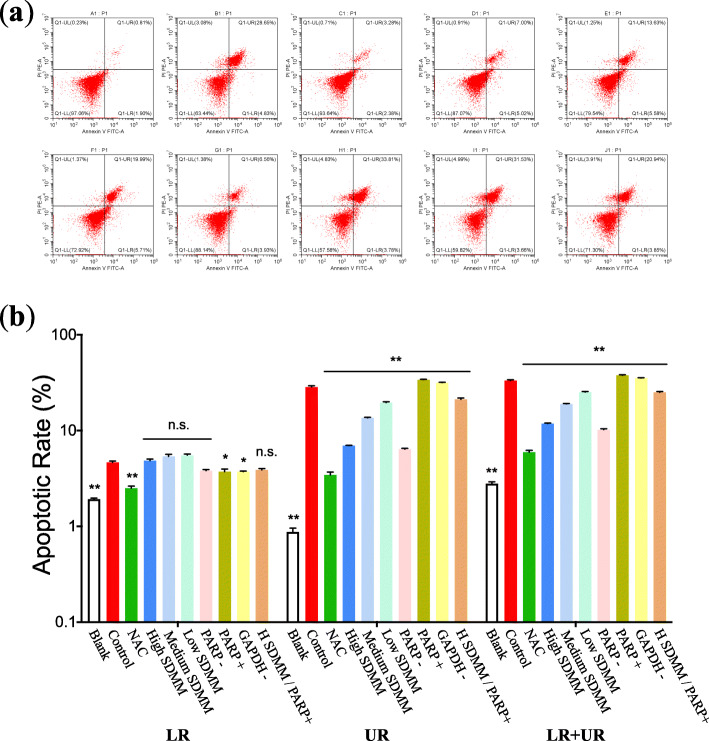
The apoptotic rate of HRMVPs (*n* = 3). **a** displays one representative cytometry plot of each group in order. LR stands for the early-stage apoptotic rate and UR represents the lateperiod apoptotic rate. As shown in (**b**), the early-stage apoptotic rate of *Control* group is apparently higher than that of *Blank*, *NAC*, *PARP +*, and *GAPDH-* group (*P*<0.05); However, there is no significant difference among *Control*, *High SDMM*, *Medium SDMM*, *Low SDMM*, *PARP -*, and *High SDMM/PARP +* group (*P*>0.05). When it comes to the late-phase apoptosis, the apoptotic rate of *Control* group is obviously higher than other nine groups, same with the total apoptotic rate (LR plus UR) (*P*<0.01). (**: versus *Control* group, *P*<0.01; *: versus *Control* group, *P*<0.05; n.s.: versus *Control* group, *P*>0.05)

**Fig. 12:**
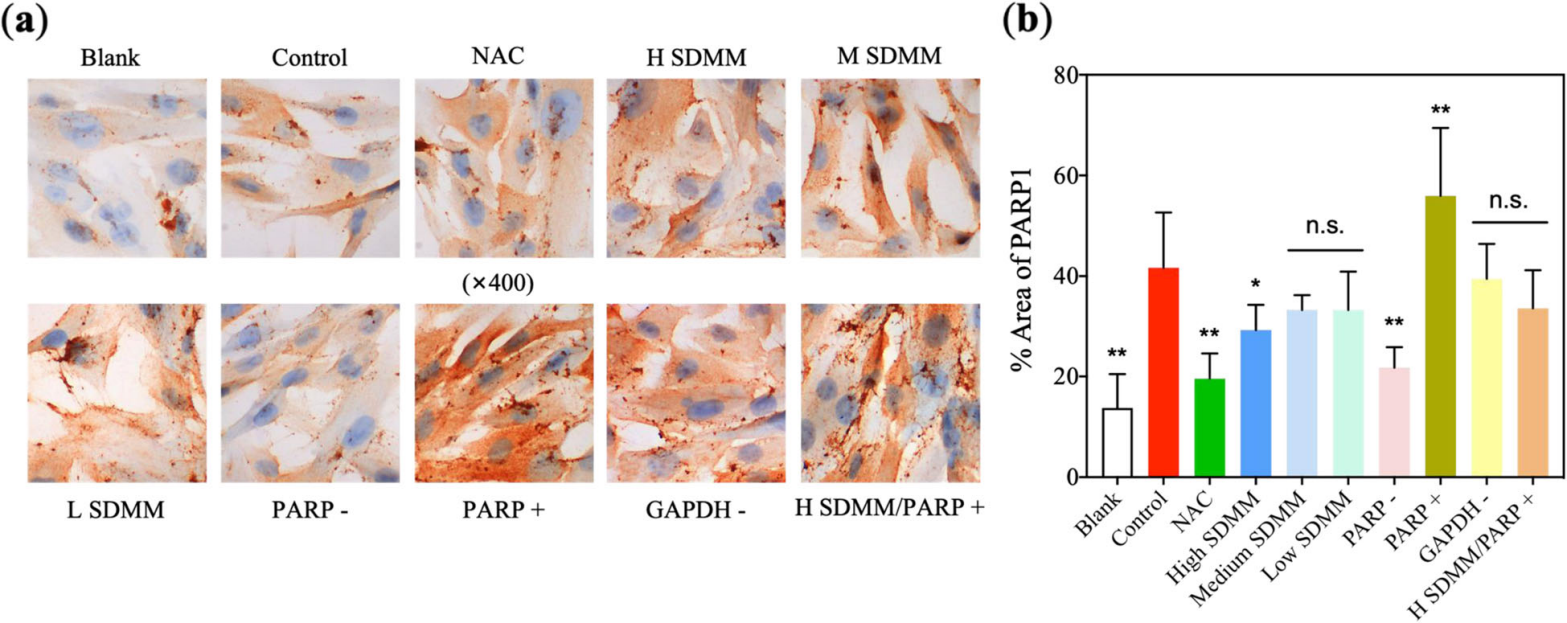
ICC of PARP1. ICC-analysis plugin of ImageJ was performed to quantify ICC results (**b**) (*n* = 3). PARP1 expression of *Control* group is obviously higher than *Blank*, *NAC*, *H SDMM*, and *PARP-* group (*P*<0.05), conversely lower than *PARP +* group (*P*<0.01) (**: versus *Control*, *P*<0.01; *: versus*Control*, *P*<0.05; n.s.: versus *Control*, *P*>0.05)

Flow cytometry was performed to determine the apoptotic rate of HRMVPs (Fig. 11) and the expression of apoptosis regulators, such as BCL2 and PARP1, was detected through RT-qPCR, WB, and ICC (Fig. 8a, b, Fig. 9d, e, and Fig. 12). The apoptosis rate, especially advanced apoptosis rate, was obviously increased after HRMVPs being incubated with H_2_O_2_, which was alleviated by SDMM capsule and PARP inhibitor, however, exacerbated by PARP activation and GAPDH inhibitor. Whereas, both SDMM capsule and PARP inhibitor showed no effect on early apoptosis (Fig. 11b). To our surprise, the total apoptosis rate of HRMVPs incubated with high concentration SDMM capsule plus PARP activation was lower than the model control group (*P*<0.01), indicating the powerful anti-apoptotic potential of high concentration SDMM capsule which might attenuate the side effect of PARP activation.

Moreover, both mRNA and protein expression of BCL2 of cells incubated with H_2_O_2_ was statistically lower than the *Blank* group, which could be ameliorated by NAC, high concentration SDMM, and PARP inhibitor, however, exacerbated by PARP activation (Fig. 8a, b, and Fig. 9d). In contrast, the expression of PARP1 of the *Control* group was obviously higher than *Blank* group (*P*<0.01) and NAC, high concentration of SDMM, and PARP inhibitor were found to downregulate both mRNA and protein expression of PARP1 (Fig. 8a, b, Fig. 9e, and Fig. 12). It’s well known to us that BCL2 is an apoptosis regulator inhibiting apoptosis through the preservation of mitochondrial membrane integrity or binding to and activating pro-apoptotic proteins. Therefore, we draw a conclusion that oxidative stress could induce increased apoptosis to HRMVPs, which could be ameliorated by high concentration SDMM and PARP1 was involved in promoting apoptosis.

#### Oxidative stress triggered downregulation of GAPDH

Both mRNA and protein expression of GAPDH was significantly downregulated after HRMVPs were incubated with 0.5 mM H_2_O_2_, which could be improved by NAC, high concentration SDMM capsule. However, PARP inhibitor could upregulate GAPDH expression by the contrary. (Fig. 8a, b, and Fig. 9f).

## Discussion

In this study, we have investigated the mechanism of SDMM capsule, a kind of Chinese patent medicine, on oxidative stress-induced apoptosis of human retinal microvascular pericytes through downregulating PARP1 and activating GAPDH expression. Research of Chinese herbal medicine, especially Chinese herbal formula, always suffers difficulties of multi components, multi targets, and multi pathways. Therefore, a network pharmacology analysis was performed as a prediction to guide this study. Only 36 kinds of components of SDMM capsule absorbed into plasma, acquired through the testing plasma of rats gavaged with SDMM capsule on our own, were collected to accomplish targets prediction, which ensured high reliability of the prediction (Table 1). Afterwards, 129 kinds of potential targets of SDMM capsule on treating DR were acquired, including PARP1, GAPDH, VEGFA, SOD, NOS, and ATK1 (Fig. 1). Moreover, PARP1 and GAPDH were found implicated in densely connected network with powerful potential in oxidative stress process, reactive oxygen species metabolic process, apoptotic signaling pathway, nitric-oxide synthase regulator activity, insulin receptor binding, glucose transmembrane transporter activity, growth factor binding, and phosphatase binding through enrichment analysis [20]. What’s more, PARP1 and GAPDH were both amongst the hub network during visualization and analysis by the BisoGenet plugin (Fig. 3). All those lay a foundation for us to research SDMM capsule on treating oxidative stress-induced apoptosis involved in PARP1 and GAPDH. Because of the critical role of apoptosis of endothelial pericytes in the early pathogenesis of DR, we pretended to pay our attention to the effect of SDMM capsule on oxidative stress-induced apoptosis of pericytes involved in the PARP/GAPDH pathway. Eventually, this prediction was verified through in vitro research.

Diabetic retinopathy (DR) remains a difficulty threatening thousands of millions of people worldwide, which is considered as the consequence of multifactorial interactions, such as gene, environment, and diet. Complex and elusive are the mechanisms of DR. Regular controlling blood glucose and early intervention of DR are thought to be effective methods. Anti-VEGF therapies have achieved a satisfying effect on attenuating diabetic macular edema (DME) and reducing fundus neovascularization. However, those approaches haven’t killed DR. Much attention has been devoted to revealing mechanisms of DR and developing new therapies for it. Several vital factors, such as apoptosis of endothelial pericytes, blood-retinal barrier (BRB) destruction, dysfunction of retinal vascular endothelium resulting in angiogenesis, have been found involved in the pathogenesis of DR [7, 21]. More and more evidence has validated overproduction of ROS affects cellular homeostasis, thereby, resulting in disease including DR [22]. Oxidative stress results from the ubiquitous expression of ROS, contributing to the pathogenesis of DR. Our group has been devoted to Chinese medicine research involved in DR. SDMM capsule, consist of *Ligustrum lucidum* Ait.*, Eclipta prostrata* (L.) L., *Cornus officinalis* Sieb. et Zucc.*, Dioscorea polystachya* Turczaninow, *Salvia miltiorrhiza* Bunge, *Panax notoginseng* (Burkill) F. H. Chen ex C. Chow & W. G. Huang, *Paeonia suffruticosa* Andr., *Alisma plantago-aquatica* L., *Poria cocos* (Schw.) Wolf.*, Smilax glabra* Roxb., *Achyranthes bidentata* Blume, is based on Chinese ancient prescriptions named *Liuwei Dihuang* Pill and *Erzhi* Pill. It showed potential capacity on alleviating symptoms resulting from DM in the perioperative period and our previous in vivo research found SDMM capsule was capable of inhibiting the overproduction of ROS of diabetic rats.

Pericytes exposed to medium containing 0.5 mM H_2_O_2_ for 4 h was performed as an oxidative-damage model in in vitro study. The extraordinarily attenuated activity and elevated apoptosis rate as well as overexpression of ROS were observed after HRMVPs were exposed to H_2_O_2_. It’s universally known that ROS is referred to as numerous oxygen-related species of high chemical reactivity. Oxidative stress (OS) resulting from overexpression of ROS is a triggering mechanism for apoptosis. As the retina is wealthy in polyunsaturated lipid membranes, it is especially sensitive to ROS. And compelling evidence indicates the presence of ROS in the vasculature has historically been regarded as detrimental and a consequence of abnormal generation and/or incapability of antioxidant systems and endogenous reductant to scavenge these reactive species. Consistently, SOD, an enzyme providing cellular defense against ROS, has been found downregulated by HRMVPs after being incubated in the medium containing H_2_O_2_ for 4 h. Whereas, these side effects evoked by OS can be alleviated by PARP inhibitor and SDMM capsule in a concentration-dependent way.

NO is quite a vital intercellular messenger and can lead to vasodilation, decreased vascular resistance, increased blood flow, inhibition of platelet aggregation and adhesion, reduced smooth muscle proliferation, and inhibition of leukocyte adhesion and transmigration under physiological condition. The specific effect of NO relies on the generated quantity and involved NOS isoforms. In general, both eNOS and nNOS can be expressed under physiological conditions and NO derived from eNOS and nNOS is of importance for maintaining ophthalmic blood flow and reducing infarct size. In contrast, iNOS is induced in vasculature under pathological situations, such as OS and inflammation, however, usually absent in vasculature in physiological circumstances. In our study, NO concentration of HRMVPC supernatant was found to be decreased after was exposed to H_2_O_2_, and the expression of eNOS, which is originally thought to be expressed mainly in endothelial cells (ECs) and was confirmed to be generated in pericytes likewise, is correspondingly downregulated.

Similarly, VEGF and ET-1, initially known to be expressed in ECs, were also found expressed by HRMVPs. VEGF is a well-known molecule physiologically required for regulating the assembling and proliferation of ECs during angiogenesis as well as for their survival and maintenance throughout the lifetime of the vasculature. Whereas, various pathological conditions, such as OS, inflammation, and ischemia, evoked during the development of DR tend to upregulate the expression of VEGF, thereby, deviate VEGF from its physiological role to numerous pathological damages. Among those damages, increasing the expression of pro-apoptotic proteins and decreasing the expression of anti-apoptotic proteins were discovered in this study.

BCL 2, an apoptosis suppressor gene, is found sharply downregulated by HRMVPs incubated in the medium containing H_2_O_2_. On the contrary, PARP1, the major PARP isoform, was obviously upregulated and the apoptosis rate of HRMVPs incubated with PARP1 inhibitor was decreased in contrast to pericytes incubated with PARP1 activation, demonstrating the pro-apoptotic characteristic of PARP1. PARP, a family of an enzyme involved in numbers of cellular processes such as programmed cell death, DNA repair, and genomic stability, can promote the diabetes-induced death of retinal microvascular cells and early lesions of DR [23]. Accelerated death of capillary cells is believed to be the major cause of acellular capillary formation.

Notably, inhibition of PARP1 can result in overproduction of GAPDH, and activation of PARP1 brings about downregulation of GAPDH in contrast (Fig. 9f). Based on the discovery mentioned above, we draw the conclusion that overexpression of ROS stimulates oxidative damage and triggers pericytes apoptosis through activating PARP1 and downregulating GAPDH. Whereas, SDMM capsule, especially high-concentration, show anti-apoptotic capacity through activating endogenous antioxidant enzymes such as SOD, thereby scavenging overproduction of ROS, and inhibiting expression of pro-apoptotic gene PARP1, and upregulating expression of anti-apoptotic gene BCL2 as well as GAPDH.

## Conclusions

Oxidative stress can induce apoptosis of pericytes. Whereas, SDMM capsule can attenuate oxidative stress induced-apoptosis through downregulating PARP1 and upregulating GAPDH. Moreover, inhibiting PARP1 expression can inversely upregulate GAPDH expression.

## Supplementary Information

**Additional file 1.**

## Data Availability

All data generated or analyzed during this study will be available from the corresponding author upon reasonable request.

## Abbreviations

PARP: Poly (ADP- ribose) polymerase
GAPDH: Glyceraldehyde 3-phosphate dehydrogenase
ROS: Reactive oxygen species
RMPs: Retinal microvascular pericyte cells
DM: Diabetes mellitus
DR: Diabetic retinopathy
OS: Oxidative stress
SOD: Superoxide dismutase
UPLC-Q-TOF·MS: Ultra-performance liquid chromatography-quadrupole time of flight mass spectrometry
SDMM: Shuangdan mingmu
PPI: Protein-protein interaction
KEGG: Kyoto encyclopedia of genes and genomes
GO-BP: Gene ontology biological process
GO-CC: Gene ontology molecular functions
GO-MF: Gene ontology cellular components
HRMVPs: Human retinal microvascular pericytes
TC0: Maximum atoxic concentration
NAC: N-acetyl-L-cysteine
NOS: Nitric oxide synthase
nNOS: Neuronal nitric oxide synthase
eNOS: Endothelial nitric oxide synthase
iNOS: Inducible nitric oxide synthase
ET1: Endothelial-1
VEGF: Vascular endothelial growth factor
BCL-2: B cell lymphoma/leukemia 2
WB: Western blotting
CCK8: Cell counting kit 8
ICC: Immunocytochemistry
NO: Nitric oxide
ATK1: Serine/threonine protein kinase 1
DME: Diabetic macular edema
BRB: Blood-retinal barrier
ECs: Endothelial cells
OD: Optical density
Ps: Phosphatidylserine
PI: Propidium iodide
DCFH- DA: 2′,7′-Dichlorodihydrofluorescein diacetate
