# Supplementary material for: Effect of Shuangdan Mingmu capsule, a Chinese herbal formula, on oxidative stress-induced apoptosis of pericytes through PARP/GAPDH pathway
Source: BMC Complement Med Ther. 2021 Apr 10;21:118. doi: 10.1186/s12906-021-03238-w (PMC8037833; doi:10.1186/s12906-021-03238-w)

Uncropped WB

1, 2, 3, 4, 5, 6, 7, 8, 9, and 10 respectively represents group A, group B, group C, group D, group E, group F, group G, group H, group I, and group J, in accordance with those in manuscript.


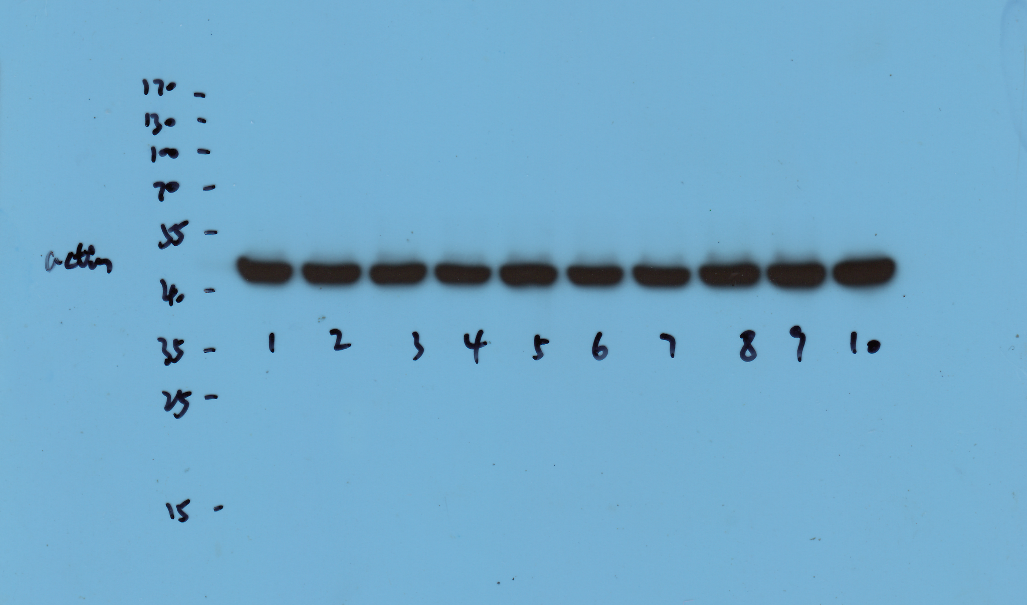

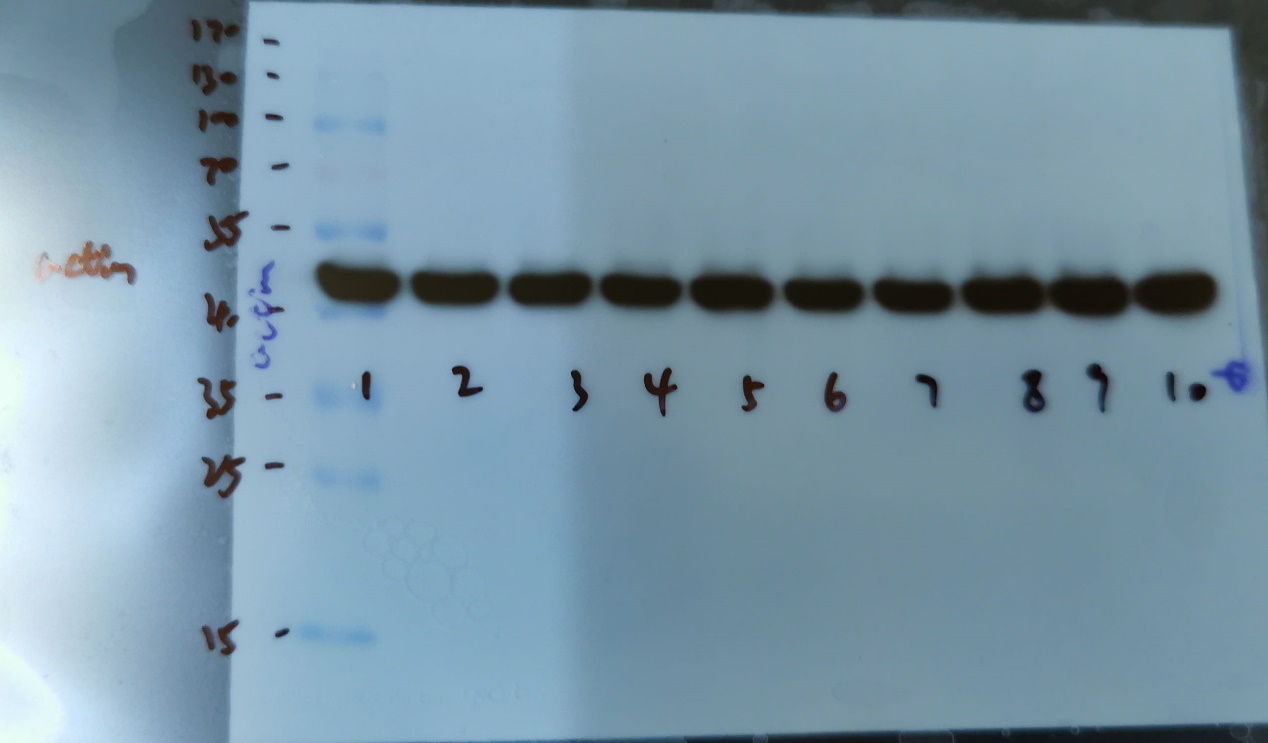


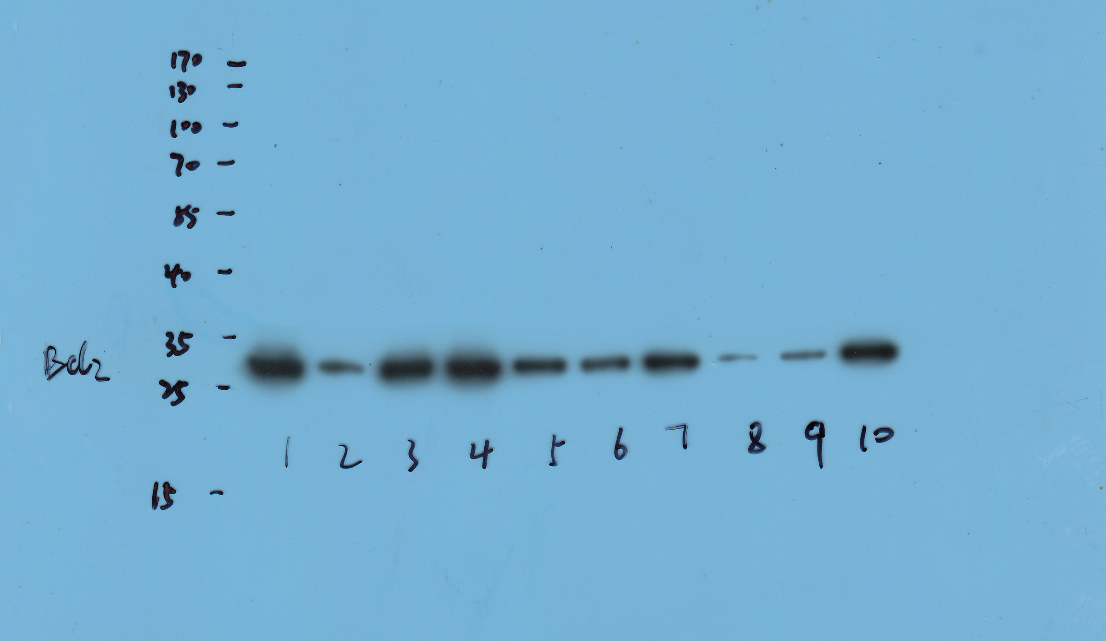

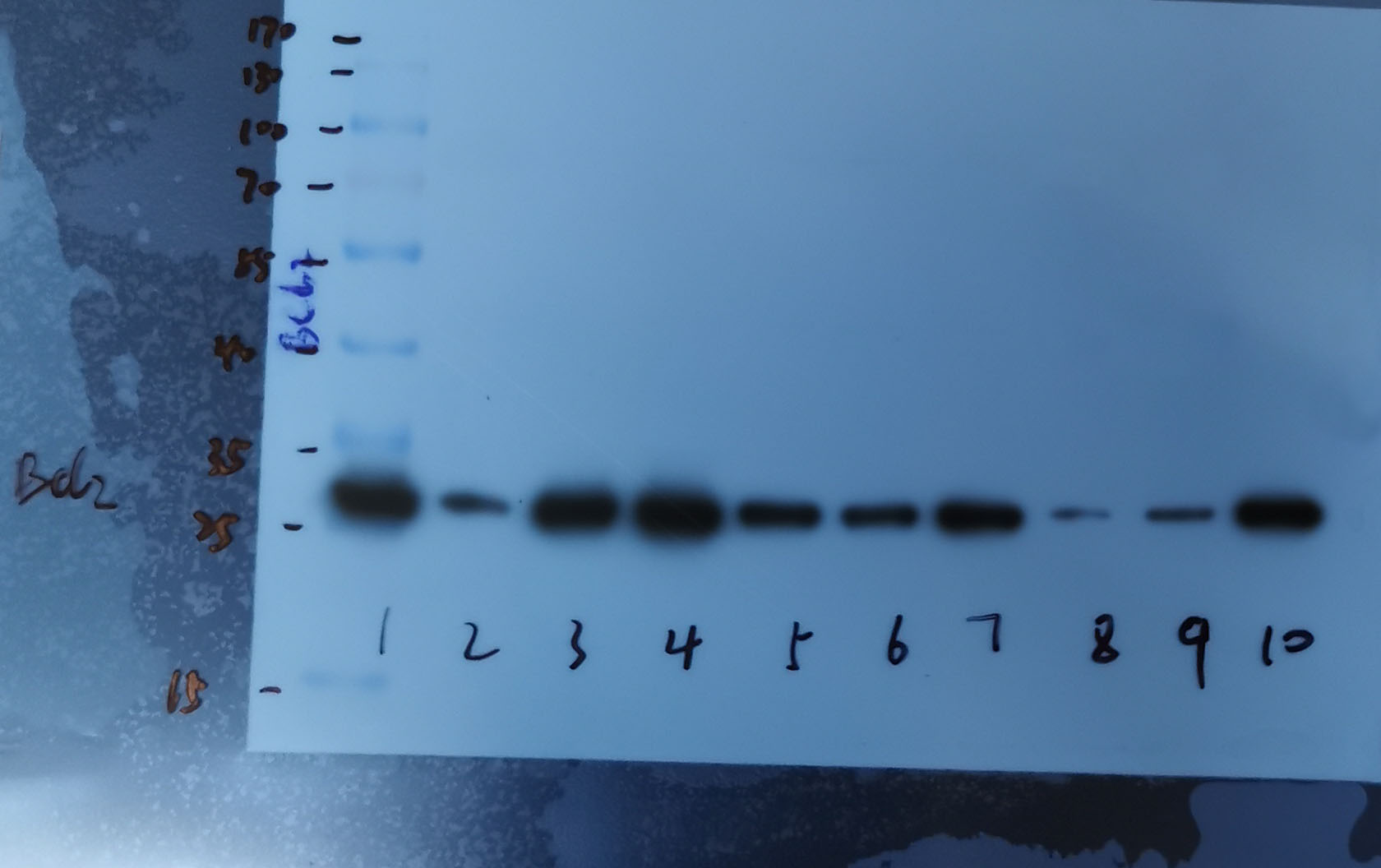


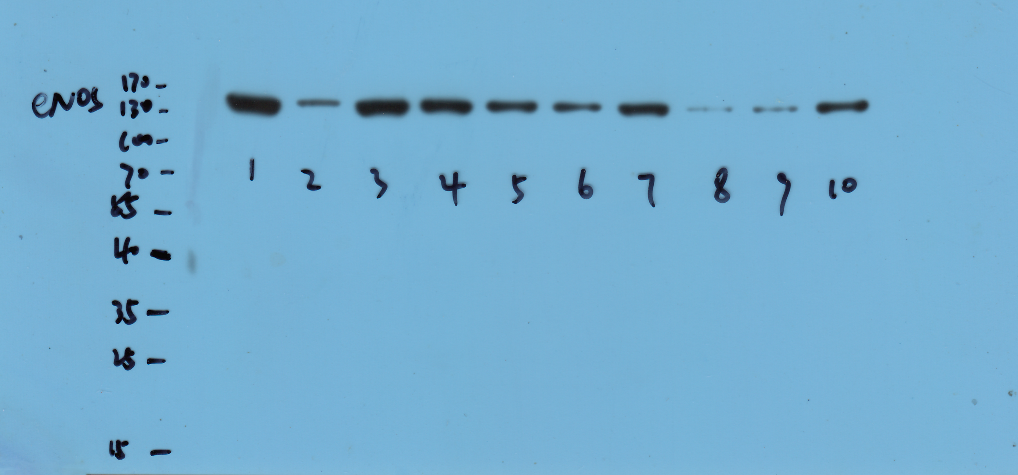

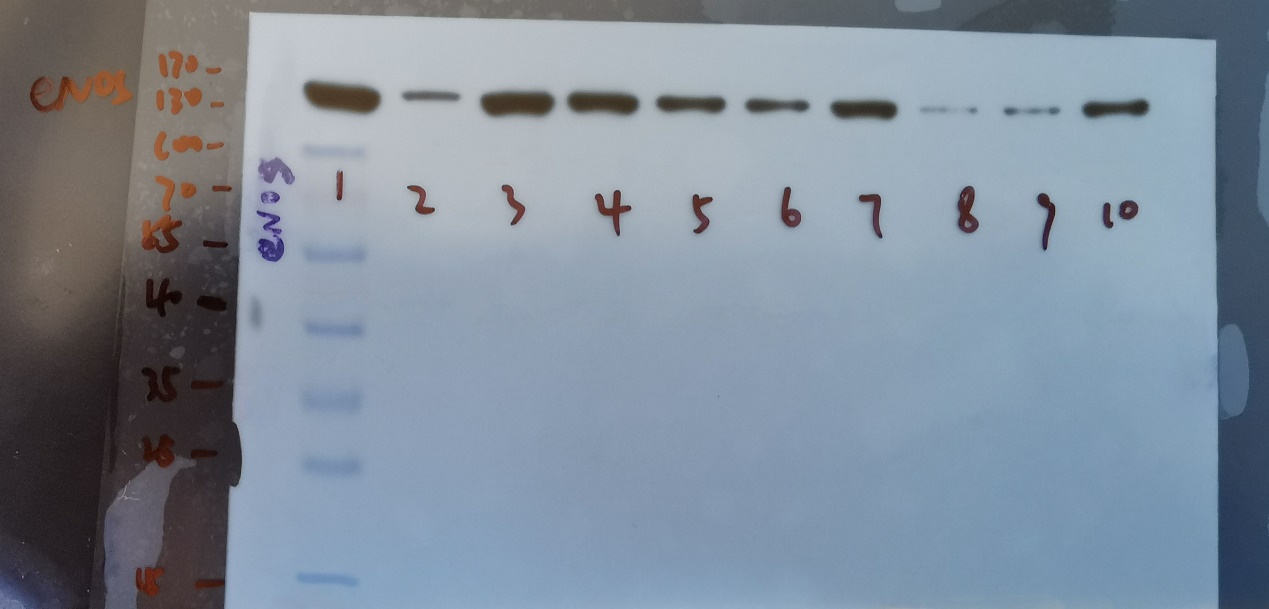


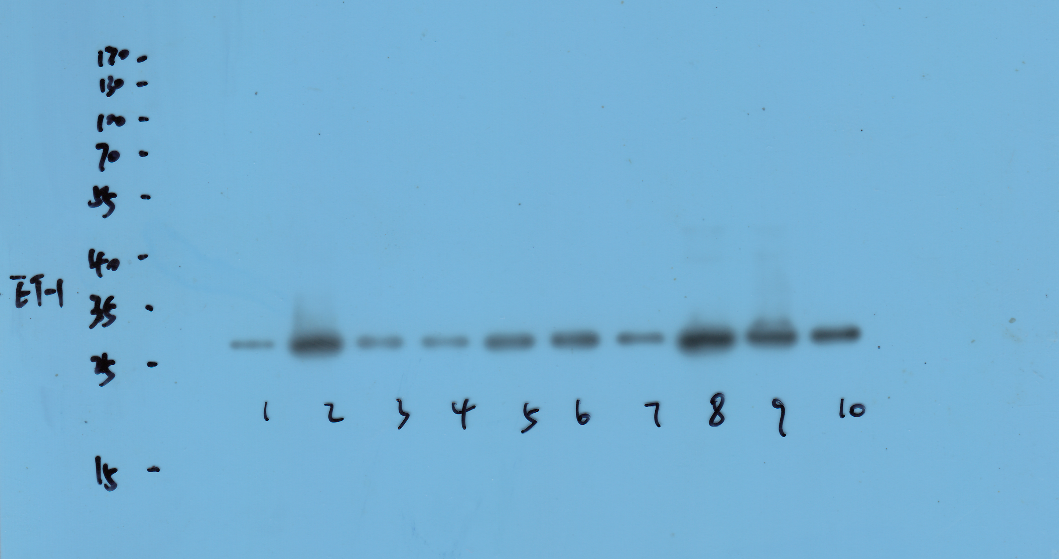


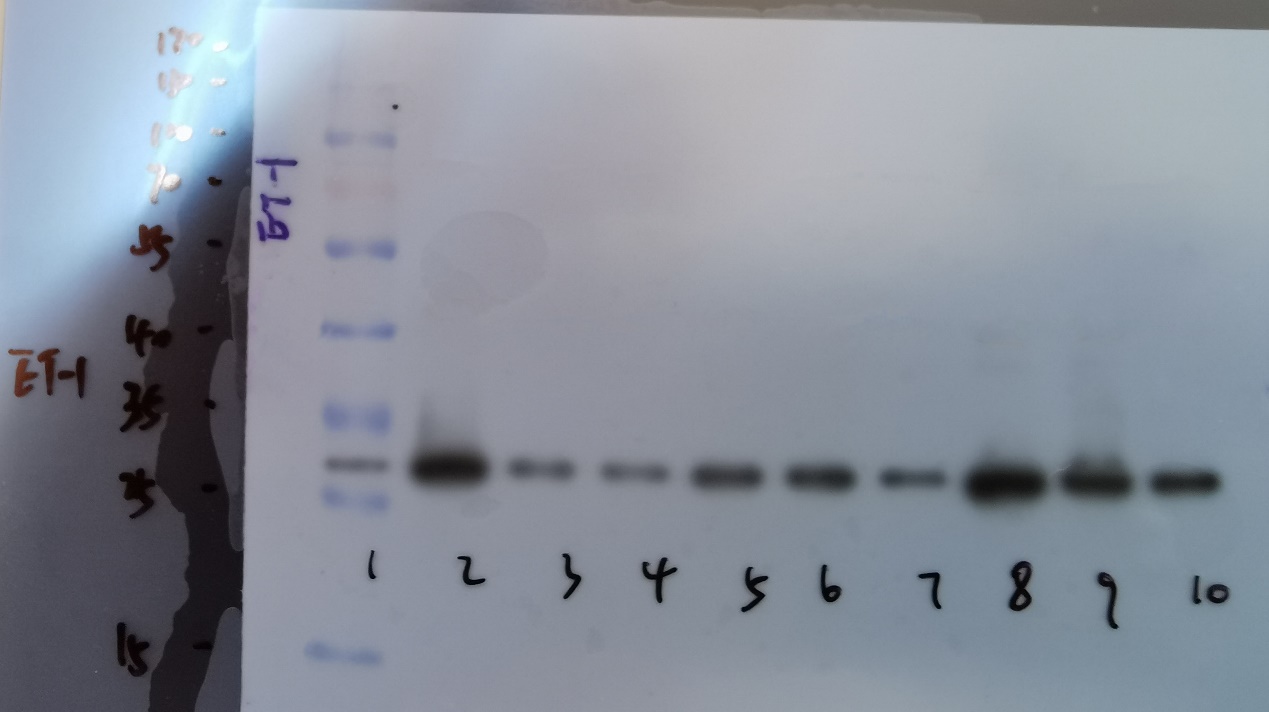


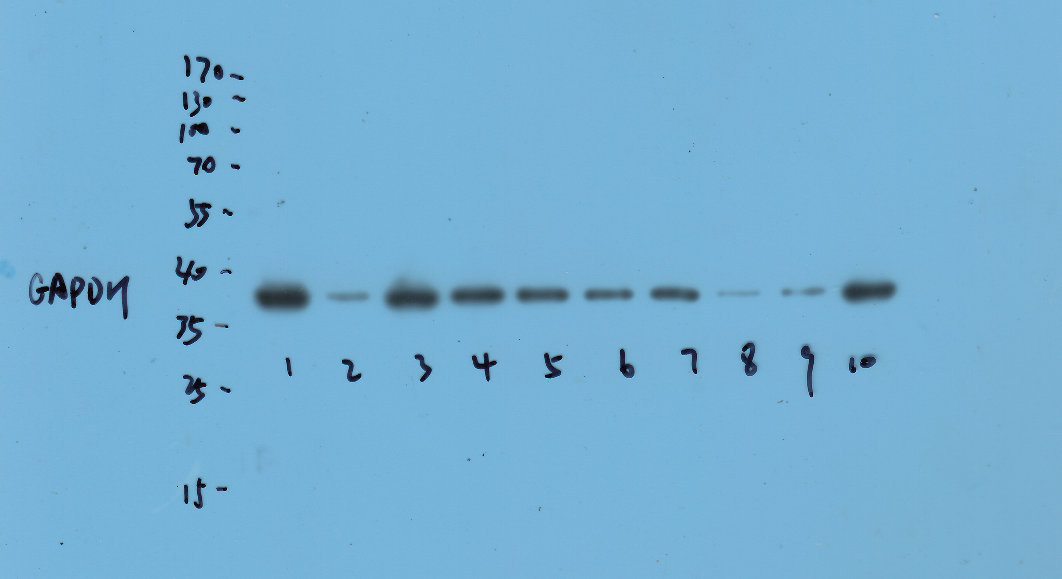


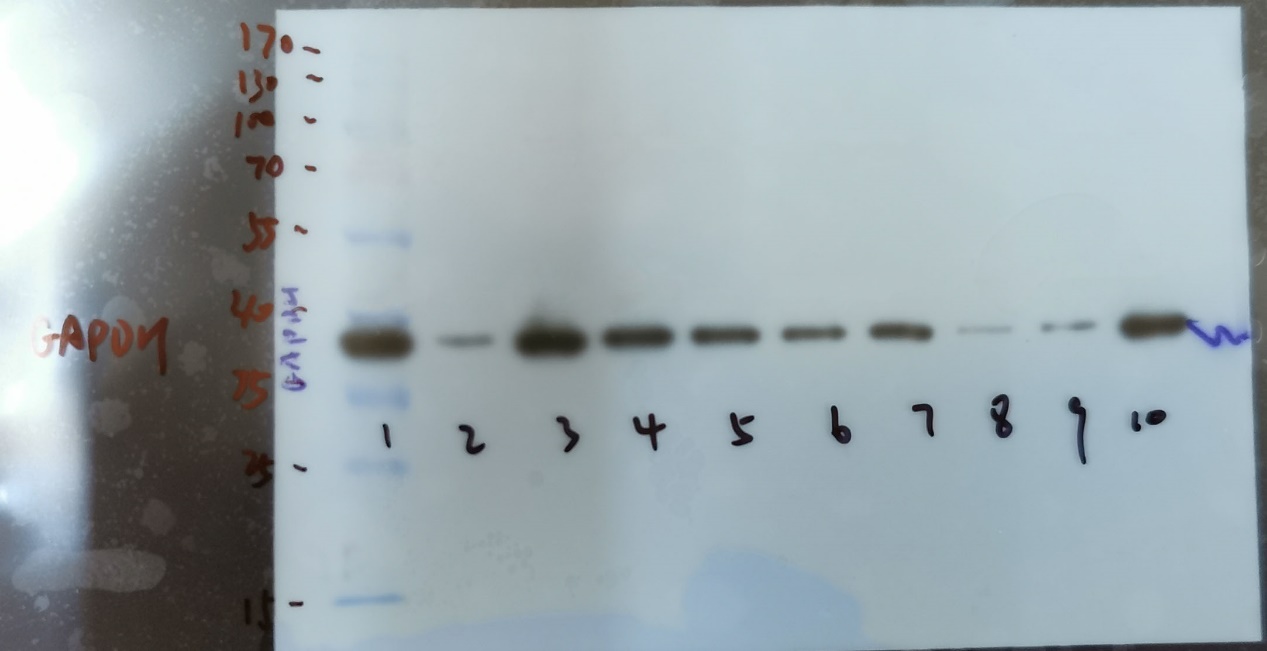


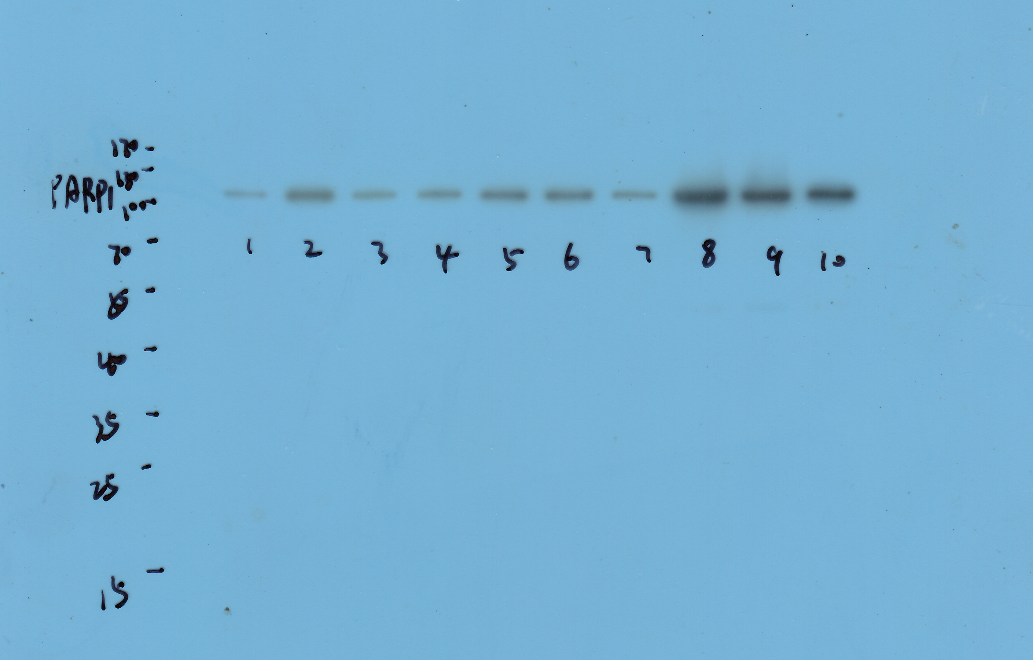


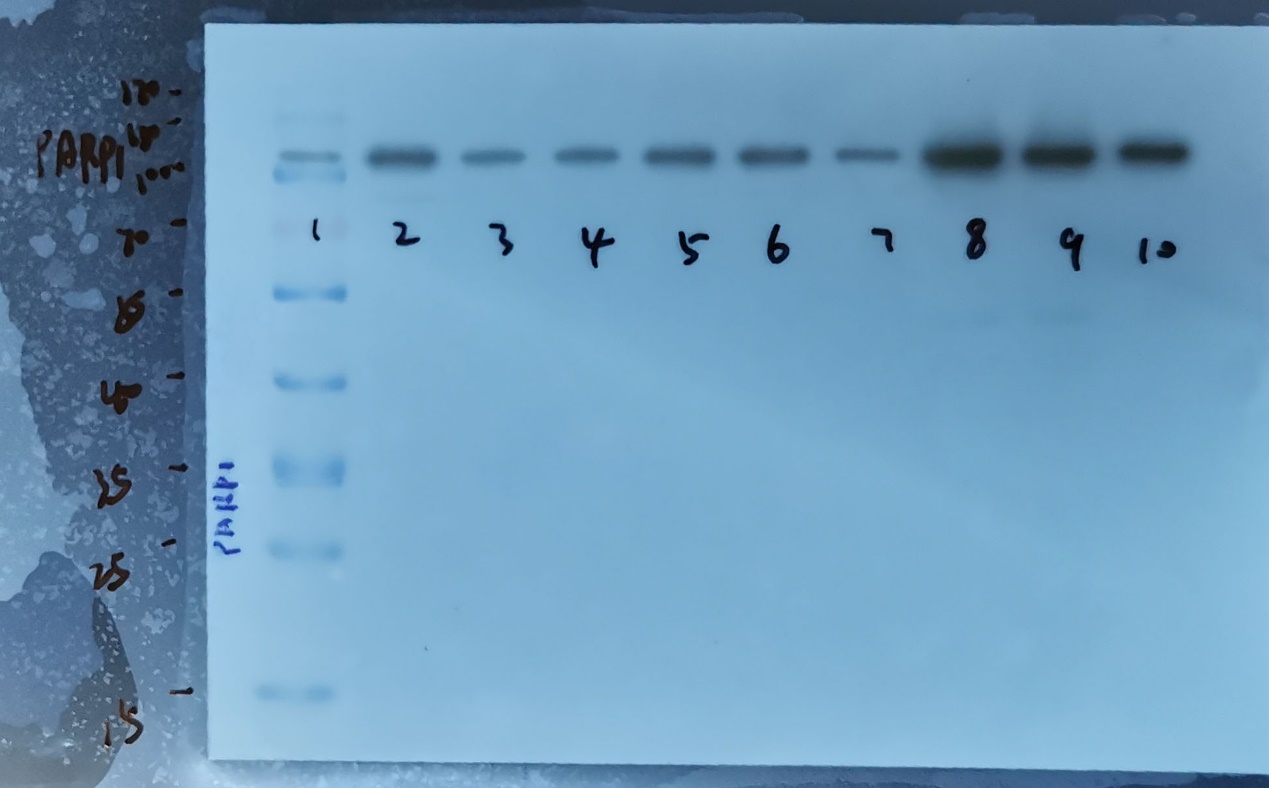


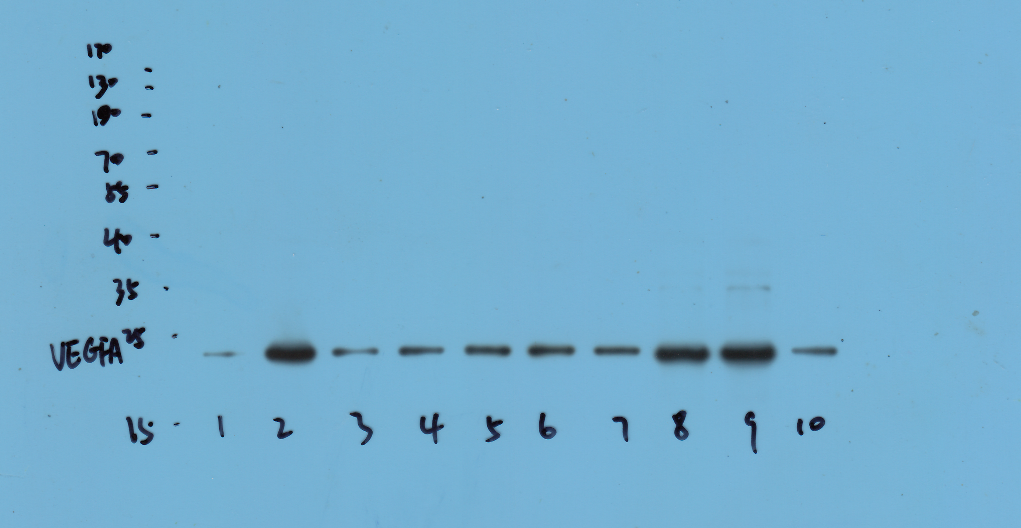

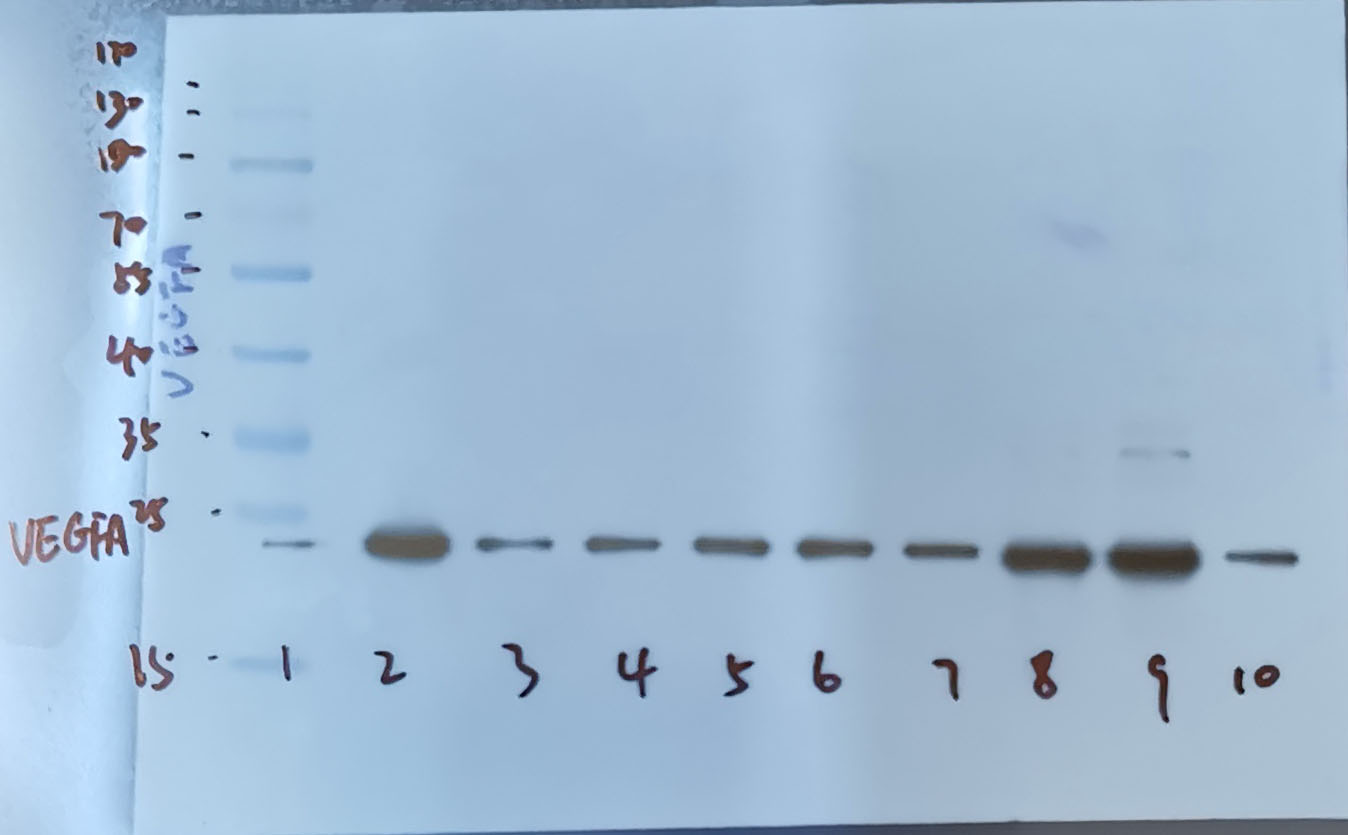

Supplement: Supplementary file 1 — Additional file 1. [file 12906_2021_3238_MOESM1_ESM.docx]
